# Forces Driving a Magic Bullet to Its Target: Revisiting the Role of Thermodynamics in Drug Design, Development, and Optimization

**DOI:** 10.3390/life12091438

**Published:** 2022-09-15

**Authors:** Conceição A. Minetti, David P. Remeta

**Affiliations:** Department of Chemistry and Chemical Biology, Rutgers—The State University of New Jersey, Piscataway, NJ 08854, USA

**Keywords:** drug discovery, drug-target interactions, thermodynamic binding signatures, isothermal titration calorimetry, lead optimization, fragment-based drug discovery, drug-like properties, enthalpic efficiency, group efficiency, lipophilic efficiency

## Abstract

Drug discovery strategies have advanced significantly towards prioritizing target selectivity to achieve the longstanding goal of identifying “magic bullets” amongst thousands of chemical molecules screened for therapeutic efficacy. A myriad of emerging and existing health threats, including the SARS-CoV-2 pandemic, alarming increase in bacterial resistance, and potentially fatal chronic ailments, such as cancer, cardiovascular disease, and neurodegeneration, have incentivized the discovery of novel therapeutics in treatment regimens. The design, development, and optimization of lead compounds represent an arduous and time-consuming process that necessitates the assessment of specific criteria and metrics derived via multidisciplinary approaches incorporating functional, structural, and energetic properties. The present review focuses on specific methodologies and technologies aimed at advancing drug development with particular emphasis on the role of thermodynamics in elucidating the underlying forces governing ligand–target interaction selectivity and specificity. In the pursuit of novel therapeutics, isothermal titration calorimetry (ITC) has been utilized extensively over the past two decades to bolster drug discovery efforts, yielding information-rich thermodynamic binding signatures. A wealth of studies recognizes the need for mining thermodynamic databases to critically examine and evaluate prospective drug candidates on the basis of available metrics. The ultimate power and utility of thermodynamics within drug discovery strategies reside in the characterization and comparison of intrinsic binding signatures that facilitate the elucidation of structural–energetic correlations which assist in lead compound identification and optimization to improve overall therapeutic efficacy.

## 1. Introduction

Tracing the historical origins of drug discovery, scientists have long pursued the paradigm of *one-drug-for-one-disease* as illustrated by the pioneering studies of Paul Ehrlich, who inspired generations of medicinal chemists in the quest of identifying, modifying, and optimizing lead compounds to achieve the desired potency, selectivity, and specificity (as reviewed in [1]). For over a century, research has advanced towards prioritizing drug-target selectivity by seeking “magic bullets” amongst thousands of prospective candidates. This scenario has proven extremely challenging as the search for novel therapeutics to treat existing and emerging diseases often relies on multi-targeting, repurposing, and/or refining an approved drug [2]. In fact, growing awareness of the cellular interactome and increasing complexity of networks controlling biological processes has recently invoked a broader selectivity (i.e., shotgun) approach [3,4]. The latter explores complex diseases that often implicate multiple targets as intervening therapies. Moreover, seemingly undruggable targets that involve functionally important protein-protein interactions (PPIs) have been unraveled and the hotspots identified therein represent novel avenues for drug discovery campaigns [5]. Irrespective of the approach, nature, and/or type of validated target, drug discovery efforts continue to accelerate with the arsenal of available tools expanding dramatically to facilitate improvements in drug efficacy while minimizing off-target effects. Considering the strategies to tackle diseases of simple or complex etiology, one principle remains unvaried, namely a drug must be designed to interact with the desired target(s) specifically and elicit the appropriate response.

The studies described and reviewed herein are intended to rekindle the interest of biochemists, biophysicists and molecular biologists on the importance of employing thermodynamic information in drug design and development by assisting hit-to-lead progression and optimization while facilitating identification and/or improvement of repurposed drugs. Major health threats related to cancer, cardiovascular diseases, and neurodegeneration remain the focus of ongoing research in immunology, virology, and a host of medical fields. The recent emergence of SARS-CoV-2 as a global pandemic warrants development of effective therapeutics as a parallel path to assist immunization efforts and mitigate the impact of vaccine resistant variants. An alarming increase in bacterial resistance has challenged the research community and pharmaceutical industry to undertake immediate action by developing the next generation of novel antibiotics and strategic approaches to obviate an imminent risk with global repercussions. This review focuses on revisiting specific accomplishments achieved in drug discovery during the past two decades particularly with respect to the role of thermodynamics as an integral tool in providing unique insights that assist decision making processes in drug design, development, and optimization [6]. The acquisition and critical assessment of thermodynamic binding signatures [7,8], in conjunction with the elucidation of structural features at the molecular level, enhance our understanding of ligand-target interactions and set the stage for improvement of predictive capabilities. A combination of biological, structural, thermodynamic, and computational approaches integrated within a multidisciplinary drug discovery strategy provides the most effective and efficient route for developing future generations of improved therapeutics.

### 1.1. Scope of the Review

This review examines the utility of employing specific criteria and metrics to guide decisions regarding the screening and selection of prospective lead compounds in the drug design and development process. While biophysical approaches, such as isothermal titration calorimetry (ITC), have been utilized to bolster drug discovery efforts, there are instances in which the power of this technique has been underutilized, serving solely as an alternate tool to derive binding affinities with the information-rich thermodynamic signatures ostensibly overlooked. In the present review, we highlight studies that have specifically explored and exploited the use of energetic binding signatures to augment drug discovery strategies and discuss the wealth of information that has been gleaned towards advancing the overall design and development process. We examine and evaluate the fundamental role of thermodynamic-based metrics to assess the fate of a compound along the drug discovery pathway. The latter is accomplished in part by seeking empirical correlations between specific physicochemical properties and experimentally derived thermodynamic profiles deduced via analysis of biomolecular interactions deposited in binding databases. Towards this end, we have scrutinized the biophysical properties and binding energetics of over 800 ligand–target complexes curated from BindingDB (http://www.bindingdb.org/bind/index.jsp, accessed on 24 April 2022) [9,10], Scorpio (http://scorpio.biophysics.ismb.lon.ac.uk/scorpio.html, accessed on 24 April 2022) [11], and PDBcal [12] tabulated within a single compiled master file [13]. The utility of employing thermodynamics-based metrics benefits from the availability of such binding data, as one can evaluate general trends and/or specific correlations that may assist in drug discovery. This review concludes by acknowledging the power of energetics in elucidating fundamental aspects of biomolecular interactions and the need for accelerating development of high-throughput technologies that integrate biological, biophysical, computational, and structural approaches in a multidisciplinary strategy to advance the design and optimization of lead compounds as novel therapeutics. 

### 1.2. Acknowledging Professor Breslauer’s Contributions to Drug Discovery

This review celebrates Professor Breslauer’s seventy-fifth birthday by reflecting on his pioneering contributions towards characterizing the thermodynamic properties of biological systems and their fundamental role in accelerating drug discovery efforts. During the past four decades, Professor Breslauer and his colleagues have focused their attention on timely topics of biological relevance by conducting biophysical research studies that assisted parallel biomedical efforts to identify effective treatment strategies for a number of devastating diseases. Such efforts have culminated in characterizing the energetics of drug–DNA interactions, particularly those directed towards the treatment of cancer and related ailments [14,15,16,17,18,19], revealing insightful trends in terms of thermodynamic binding signatures [18,19,20,21,22] and yielding invaluable information content for advancing drug discovery strategies [23,24,25]. In a series of seminal studies, Professor Breslauer established a database of nearest neighbor thermodynamics for canonical DNA duplexes [26], advanced the concept of enthalpy-entropy compensation in ligand–target interactions [15] which represents a formidable challenge in drug design efforts, explored the relevance of heat capacity changes on DNA duplex energetics [27,28] and macromolecular processes [29], and characterized the thermodynamics of template-directed polymerase synthesis [30]. In collaboration with Professors Grollman and Johnson at Stony Brook University, the Breslauer research group has investigated oxidative DNA damage, which causes a myriad of diseases including cancer and neurodegeneration. These efforts have complemented biophysical studies of canonical DNA with detailed thermodynamic analysis of lesion impacts on duplex energetics [31,32,33,34,35,36,37,38,39,40,41,42] as well as lesion recognition and repair by DNA glycosylases [23,29,43,44]. While establishing the foundation required for understanding the energetic basis of complex biological systems, the Breslauer research group has focused on gene transcription regulation, repair, and replication [29,30,39,45,46]. Professor Breslauer’s impressive academic career spanning five decades is chronicled in a recent retrospective [23] as his research group continues to pursue effective therapeutic interventions for combating and treating infectious diseases such as the SAR-CoV-2 pandemic [47]. In summary, the indelible impact of Professor Breslauer’s pioneering contributions to the field of biothermodynamics and their consequential impact on advancing drug discovery strategies is gratefully acknowledged and recognized by a legion of peers.

## 2. Drug Discovery Strategies

The design, development, and optimization of prospective compounds as effective therapeutic agents require broad-based drug discovery strategies that incorporate high throughput screenings (HTS) in conjunction with multidisciplinary characterization protocols. HTS aims at generating selected hits from millions of compounds that are further optimized into chemical leads. The identification of an ideal chemical lead and its overall quality is dictated by a host of properties that extend well beyond inhibitory activities and binding affinities. These include specific pharmacokinetic properties, such as absorption, distribution, metabolism, excretion, and toxicity that are collectively termed ADMET. While deemed essential to ascertain a sustained therapeutic level upon oral administration, ADMET properties are generally more difficult to optimize than primary biological activity [48]. Considering their fundamental role in evaluating drug efficacy, complementary approaches to HTS are instrumental for monitoring the optimal predicted performance of a compound following in vivo administration. Drug discovery strategies are continually expanding capabilities in terms of both restructuring facilities to experimentally access and accelerate the production of hits [48] while intensifying the use of complementary computational approaches to expedite compound screening. During the screening process, several rules are imposed on the basis of selected criteria and/or metrics to categorize the drug-likeness of a compound. Such criteria rely on a host of properties that assist in evaluating a compound based on its desired qualities as a pharmacophore in addition to specific biological and ADMET characteristics. The criteria employed for screening potential therapeutic agents are presented and reviewed within the context of information gleaned from several publically accessible databases.

### 2.1. Fragment-Based Drug Discovery

**Figure 1:**
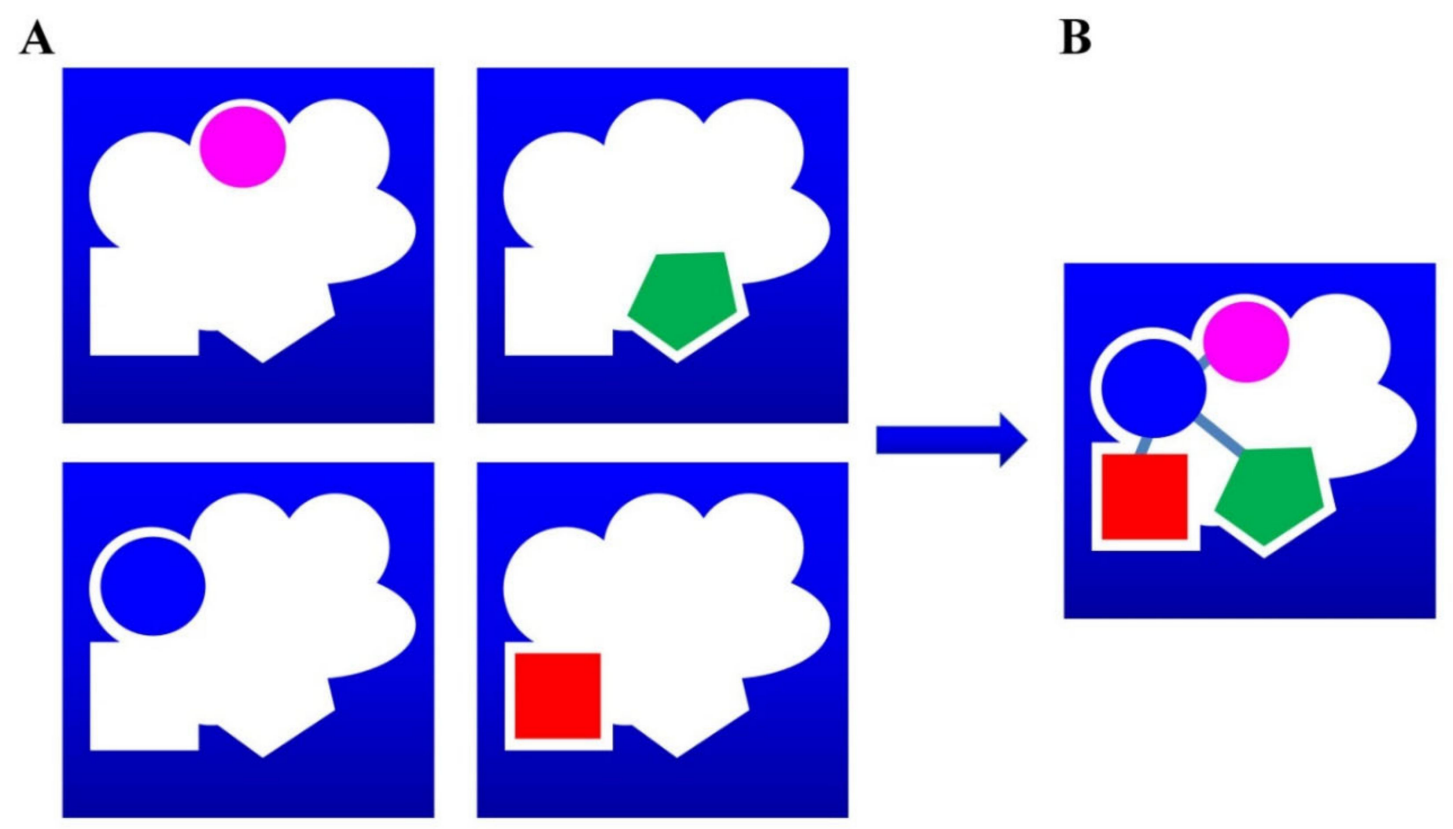
Representative linking approach commonly employed in FBDD to generate an optimized lead compound. (**A**) Several small fragments bind to the receptor within specific subpockets (as represented by blue and magenta spheres, a green pentagon, and red square). (**B**) The fragments are linked according to their spatial distribution within the binding site, which allows the resultant lead molecule to interact with a larger surface and therefore exhibit a significantly higher affinity relative to its constituent fragments [48].

The strategy of fragment-based drug discovery (FBDD) originated as a natural progression from a concept initially proposed by Jencks to characterize the binding energetics of protein–ligand complexes [49]. Invoking this empirical approach, the binding free energy of a ligand might be visualized as the sum of its component fragment interactions with specific protein sub-sites. A subsequent study employed these principles to examine the differential impact of tethered small molecules *versus* their isolated counterparts on protein-inhibitor binding energetics [50]. Significantly, the tethered ligand exhibited a dramatic enhancement in binding affinity approaching three orders of magnitude (i.e., nanomolar vs. micromolar). This proof-of-principle demonstrated the potential utility of incorporating additivity in target-directed research and effectively served as the predecessor of FBDD. A follow-up study applied this methodology towards the screening of prospective papillomavirus E2 protein inhibitors and identified ligands that bind weakly to adjacent sites with the prospect of tethering these molecules within a higher affinity compound [51]. Given the collective success of such protocols in contrast with the limitations of prior approaches, FBDD has advanced into a multidisciplinary endeavor (as reviewed in [52]) and is considered one of the preferred strategies in current drug discovery programs [53]. FBDD essentially consists of screening and identifying small molecules that bind proximal subsites in the target protein, which are subsequently optimized and the resultant fragments linked to produce higher affinity ligands with the desired biological activity/potency (as reviewed in [54]). A schematic overview of FBDD presented in Figure 1 illustrates how low affinity molecules that interact with subpockets of the binding site are subjected to biophysical screening and the optimized fragments assembled into high affinity ligands [48]. 

FBDD has been introduced as a powerful alternative and complementary technique to traditional HTS strategies for the identification of lead molecules. Several new compounds derived via FBDD have reached the clinical development stage with progressively more attention devoted towards applying this method in drug discovery protocols [55]. Fragment libraries of relatively small molecular weight compounds (~100–300 Da) are screened via a combination of structural, functional, and biophysical techniques. The initial screening process in FBDD employs X-ray crystallography and/or nuclear magnetic resonance (NMR) spectrometry to identify prospective target-fragment complexes. Subsequent characterization via biophysical and functional approaches yields the requisite fragment binding affinities and inhibition activities. Optimization of the initial hits generates a higher molecular weight ligand comprised of multiple fragments which exhibits both enhanced affinity and potency. In most successful examples of fragment optimization, discrete contacts are responsible for securing the anchoring fragments and these binding modes are preserved throughout the optimization process. Specific details regarding the various steps encompassing *hit-to-lead-to-drug*, the strategies utilized in FBDD, and optimization of the final molecules are described and reviewed elsewhere [56]. Some of the most successful applications of fragment-based methods have involved deconstructing known leads and reassembling these to generate a new chemical series with improved biological properties [57].

Despite the overall success achieved in identifying prospective lead compounds, FBDD screening methodologies often encounter challenges when the binding mode of assembled fragments within adjacent pockets are disrupted as a consequence of geometric constraints imposed by the linker [58]. This represents a potential shortcoming of FBDD as the anticipated gain in potency may be impaired by the difficulty of linking fragments to form an active compound [59]. Moreover, potential undesired physicochemical properties may compromise a drug’s performance in vivo, despite its ability to bind the target and elicit a desired effect in vitro. An experimental strategy that incorporates binding thermodynamics and prediction metrics during the early stages of FBDD may therefore prove extremely useful in terms of ensuring a successful outcome. Specifically, a rigorous thermodynamic analysis may furnish invaluable information regarding biophysical properties that are often overlooked when a molecule is evaluated solely in terms of binding affinities and inhibition constants. The utility of thermodynamics in drug design, development, and optimization is the focus of this review and discussed in subsequent sections.

### 2.2. Computational and Experimental Methods to Assess Drug Potential: Criteria and Metrics

A multitude of parameters for evaluating the quality of a molecule in terms of specific “drug-likeness” attributes have been adopted over the years and are generally considered as useful metrics to assess whether a compound fulfills basic physicochemical properties that serve as a prerequisite for subsequent development into an effective therapeutic agent. Several of the proposed metrics are better suited for a particular class of compounds, route of administration, target location, or nature of the screening protocols (as reviewed in [60]). These empirical criteria continue to evolve on the basis of advancements achieved in the design, development, and optimization of successful lead compounds. In the following sections, we discuss specific criteria and metrics that have been proposed and utilized as cut-off filters in drug discovery strategies.

#### 2.2.1. Criteria for Selecting Prospective Lead Compounds

Among commonly adopted criteria to assess the overall fitness of a molecule as a prospective drug, Lipinski’s *rule of 5* (Ro5) [61] has been widely employed to predict permeability and solubility characteristics, particularly in the case of compounds intended for *oral* administration. Computational data have assumed a significant role in providing general criteria and guidelines to determine whether a compound satisfies Ro5 stringency. In specific terms, the limits for reasonable absorption and/or permeation require a molecular weight (MW) no greater than 500, a maximum of 5 H-bond donors (HBD) and 10 H-bond acceptors (HBA), a calculated LogP (CLogP) that does not exceed 5, and the absence of toxic groups. Considered a useful parameter to analyze small molecules, LogP corresponds to the logarithm of its partition coefficient between octanol and water (i.e., Log [C_o_]/[C_w_]), and therefore represents a well-known measure of molecular hydrophobicity and/or lipophilicity [61,62]. LogP is determined to assess biological properties that are relevant to drug performance, including lipid solubility, tissue distribution, receptor binding, cellular uptake, metabolism, and bioavailability. LogP is commonly estimated via parameterization methods and the calculated values (i.e., CLogP or AlogP) are routinely used in the prediction of LogP with relatively comparable performance [63].

Although predictions are useful for evaluating related analog series, experimentally derived binding energetics and solubility measurements are deemed essential to unequivocally ascertain whether a molecule will perform as planned. Ro5 is insightful and serves as a useful guideline or extra filter in screening prospective compounds. Nevertheless, the fluidity of emerging data requires constant vigilance and updates to monitor novel classes of compounds and targets identified in the drug discovery process. The ultimate goal of achieving high potency and selectivity via application of specific metrics is paralleled by the expectation that an ideal compound operates within a reasonable therapeutic window. In this respect, basic drug-like properties must be viewed within the prism of achieving efficacy while precluding toxicity. The latter is accomplished by incorporating easily conjugated (e.g., hydroxyl, amino, carbonyl), metabolically cleaved (e.g., ester, amide), oxidizable, and excretable (e.g., methyl) groups/metabolites, all of which ensure maximum efficacy and minimal side effects (as reviewed in [64]).

**Figure 2:**
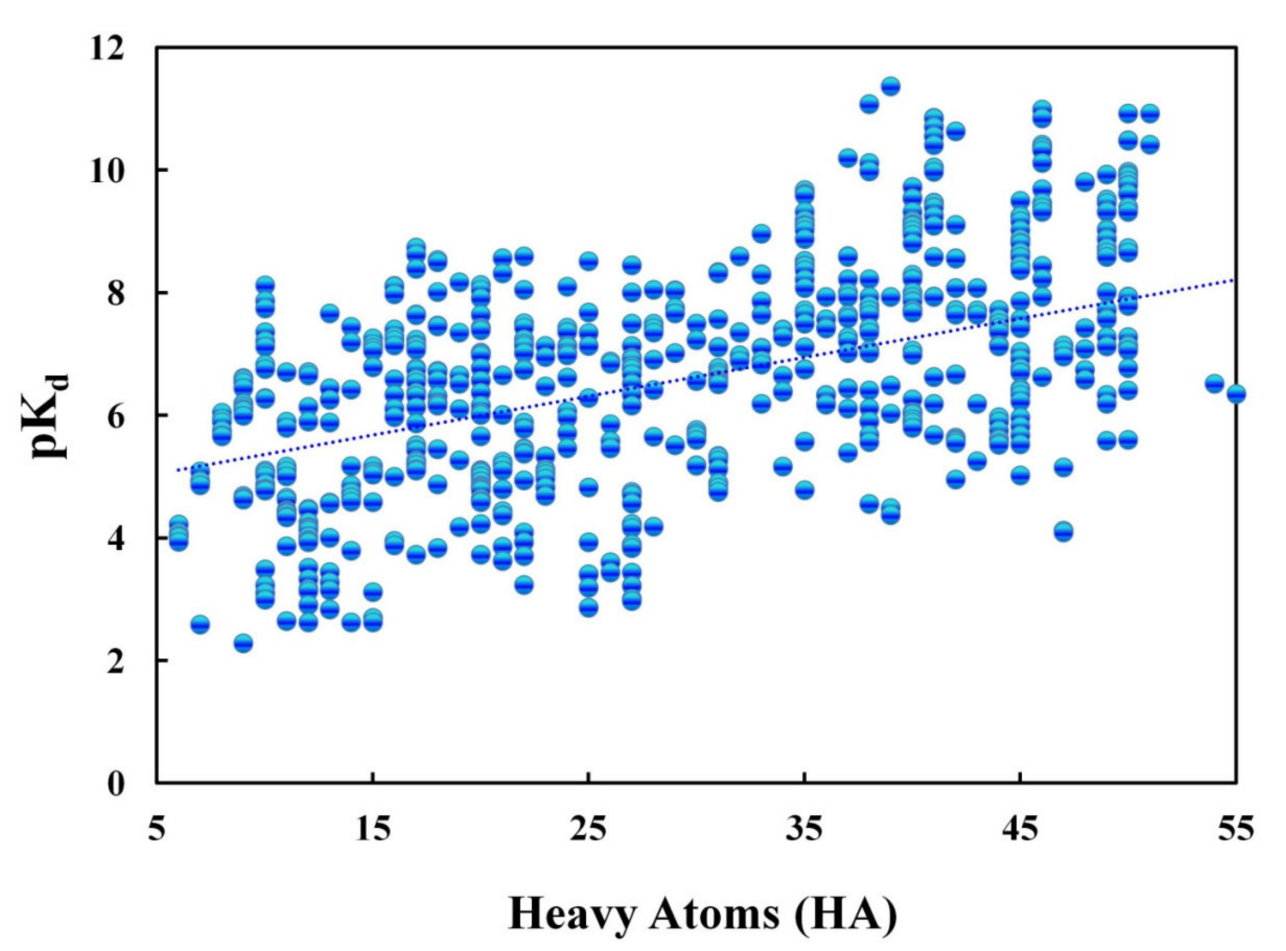
Correlation of ligand potency and molecular size deduced via pK_d_ distribution as a function of ligand heavy atom (HA) number [13].

The need for standardized metrics to predict/evaluate compound potency is justified due to the realization that screening processes are generally biased towards selecting higher MW compounds [65] given their propensity to exhibit enhanced binding affinities. An observable trend is that lead developments have progressively resulted in the selection of compounds with increased molecular weight. In fact, this screening bias is evident as ligand potency often tracks with molecular size as noted by inspection of pK_d_ distribution tendencies as a function of heavy atom (HA) number in Figure 2. Such qualitative trends are quite visible in larger datasets and particularly striking when higher potency compounds are selected for analysis [66]. On the basis of Ro5 guidelines and in view of anticipated problems associated with drug solubility and biodistribution, lower molecular weight compounds are typically the most effective therapeutic agents with an optimal MW below 400 Da. 

**Figure 3:**
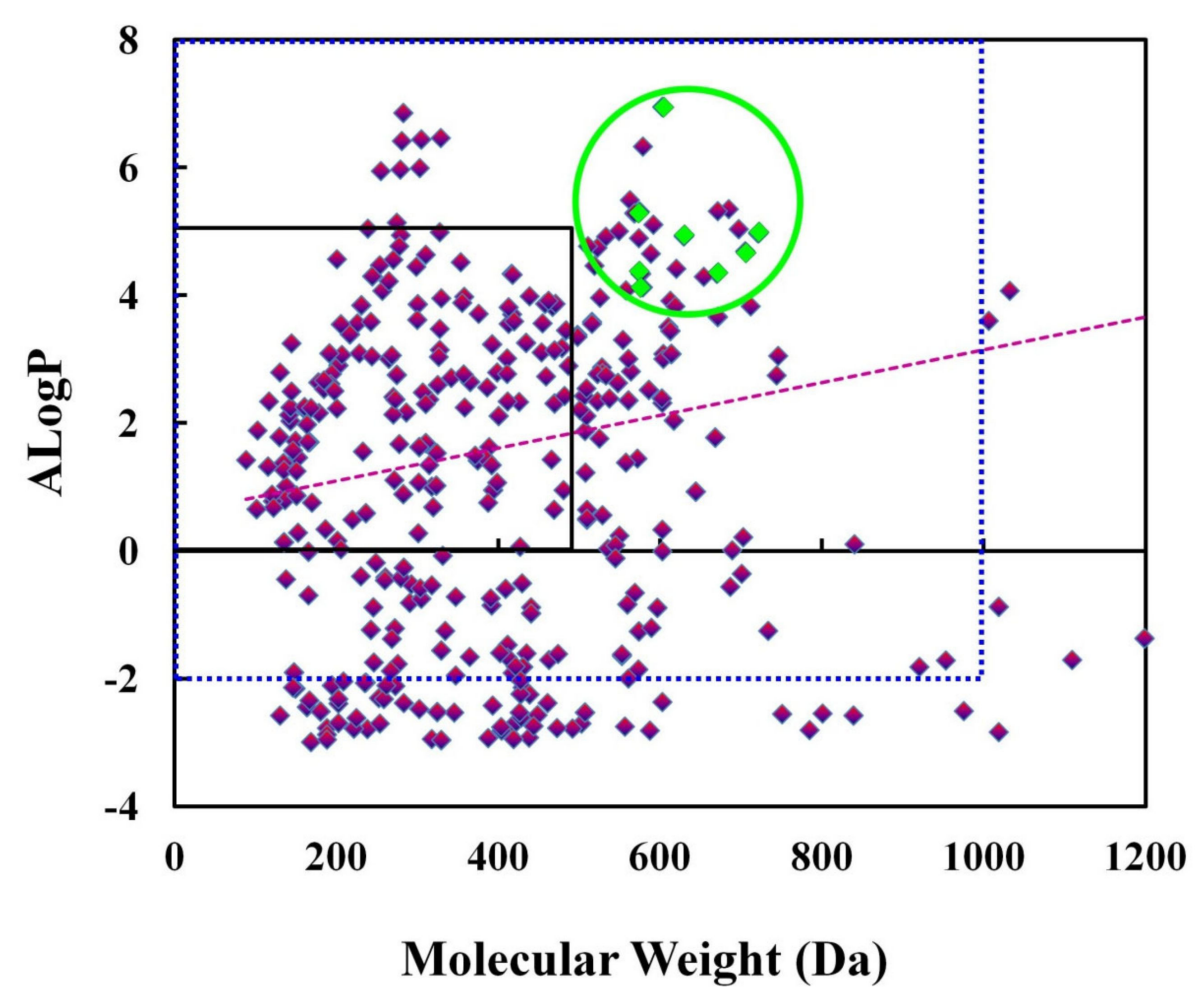
LogP (calculated as AlogP) values [13] plotted as a function of compound molecular weight (MW). The black box delineates an area within which the majority of drugs approved for oral administration are located. The blue dashed-box extends this area to higher molecular weight compounds distributed over an expanded range of LogP which contains exceptions to the rules (Log P ≤ 5 and/or MW < 500) yet results in approved drugs. The encircled green diamonds represent some of the compounds employed successfully in anti-HIV therapeutics.

A significant observation is that drug candidates and novel molecular entities (NME) tend to exhibit increased hydrophobicity, generating concerns regarding their overall biophysical properties and effectiveness [61]. Assessment of available data in terms of its conformance with Ro5 criteria can be gleaned via inspection of LogP values as a function of molecular weight. Viewing the graphical depiction in Figure 3, most of the approved oral drugs are clustered within an area represented by the black box and thereby fulfill this general rule (i.e., LogP ≤ 5). As with all metrics employed in the evaluation of an ideal drug, there are successful outcomes that do not strictly comply with stringent rules [67]. In such cases, the overall distribution extends beyond Ro5 [68] as evidenced by compounds residing in the dashed blue box (Figure 3) which still appears densely populated. In the specific example of HIV-protease inhibitors represented as light green diamonds (Figure 3), the overall molecular size is in fact greater than 500 Da and LogP values are border line Ro5 limits. 

**Figure 4:**
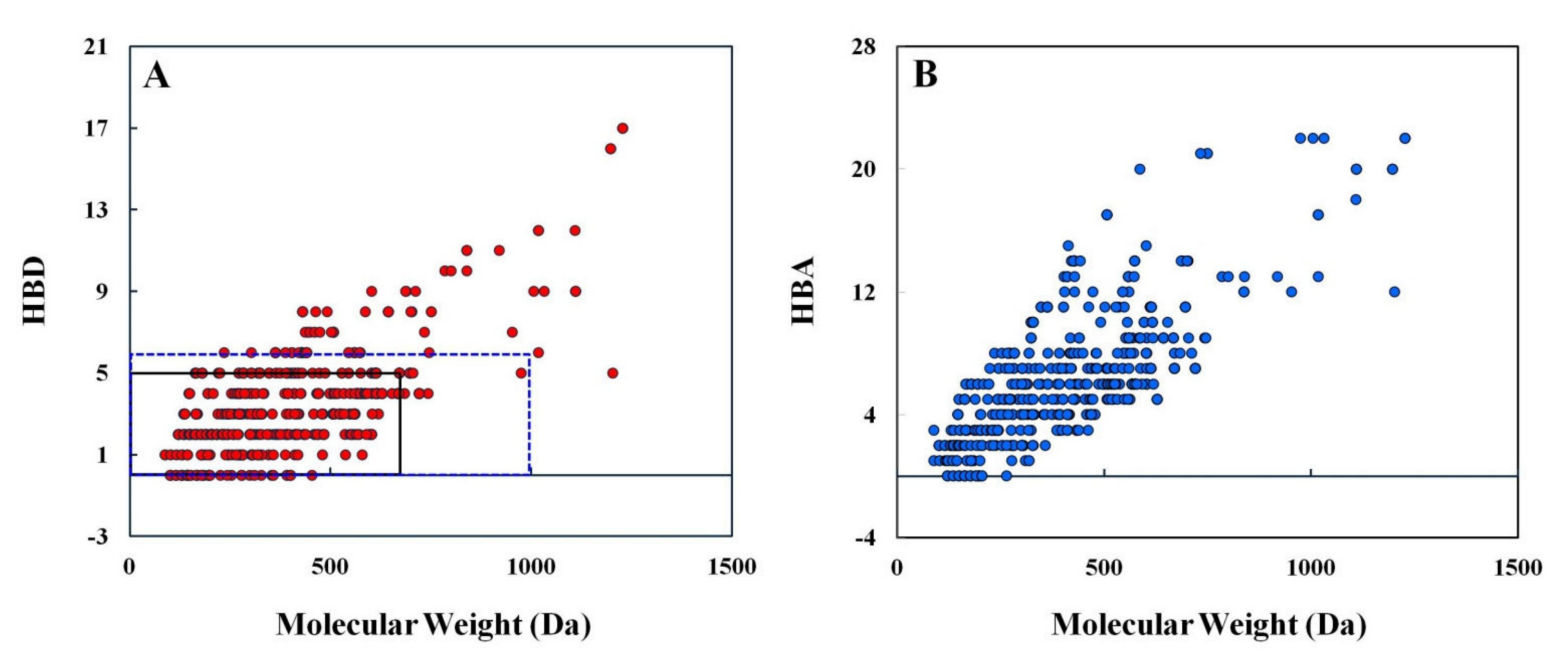
Molecular size distribution of compounds deposited in public databases [13] according to the number of (**A**) hydrogen-bond donors (HBD) and (**B**) hydrogen-bond acceptors (HBA).

Another critical issue concerning the application of Ro5 in drug design pertains to HB donors (HBD) and acceptors (HBA), the former of which has been scrutinized extensively in lead optimizations. Inspection of available data suggests that most compounds appear to abide within the limit of HBD < 5 as illustrated in Figure 4. Despite this evidence, there has been a recent upsurge in the number of drugs approved for therapeutic use that tend to violate HBD and pertinent Ro5 criteria [69,70,71,72]. In view of these developments, suggestions to expand such limits have been considered and a modified version designated as *beyond Ro5* (bRo5) proposed [69]. In retrospect, compounds harboring bRo5 chemical space already existed amongst natural products (NP), and the advent of semi-synthetic as well as total synthesis protocols has expedited the availability of such molecules [73]. Inspired by nature, an increasing number of compounds isolated from plant or marine sources have been investigated and their potential applications explored. These include biologically active defense molecules and antimicrobial peptides that have been studied and elaborated as future templates to combat the alarming rise in bacterial resistance [74,75].

Given the recent emergence of SARS-CoV-2, chemists have resorted to evaluating a myriad of novel, repurposed, synthetic, and/or NPs as prospective antiviral molecules [76]. A large number of compounds are now viewed beneath the umbrella of expanded criteria and include targets with “difficult” binding sites that are commonly designated as difficult-to-drug targets. The latter comprise large, highly lipophilic or polar, and/or flexible binding sites [77]. This category includes protein–protein interaction (PPI) inhibitors, a rapidly expanding class of compounds that are designed to disrupt the large protein-protein interface and as a consequence do not fulfill basic Ro5 criteria. Moreover, recent advances in drug discovery directed towards targets that comprise intrinsically disordered proteins (IDP), which are implicated in a host of disorders including cancer [2] and neurodegenerative diseases [78], have resulted in candidates evading traditional metrics. Unusual target sites cannot accommodate conventional therapeutics, and thus require higher molecular weight, lipophilic, or polar compounds that are more amenable to the bRo5 concept [68] or evade any set of criteria/rules that define a drug-like molecule [67].

The drug-like properties of therapeutic compounds approved for oral administration have been evaluated in terms of their molecular and physical properties [79]. The overall findings suggest that reduced flexibility, expressed in terms of rotatable bonds (i.e., ROTB ≤ 10), low polar surface area (i.e., PSA ≤ 140 Å^2^), or total hydrogen bond count (i.e., sum of HBD and HBA ≤ 12) are reasonable predictors of oral bioavailability, irrespective of molecular weight. These observations underscore the need to critically assess and balance pros versus cons when evaluating the overall degree of success exhibited by a drug, as concurrent factors such as target affinity, selectivity, permeation, and clearance may benefit from distinct or even opposing molecular properties. In this respect, the unanticipated positive impact of molecular rigidity (i.e., reduced ROTBs) has to be considered in terms of a shape that retains the ability to interact with a target, thereby enhancing activity and selectivity to bind carrier/clearance proteins and optimally permeate membranes. 

**Scheme 1:**
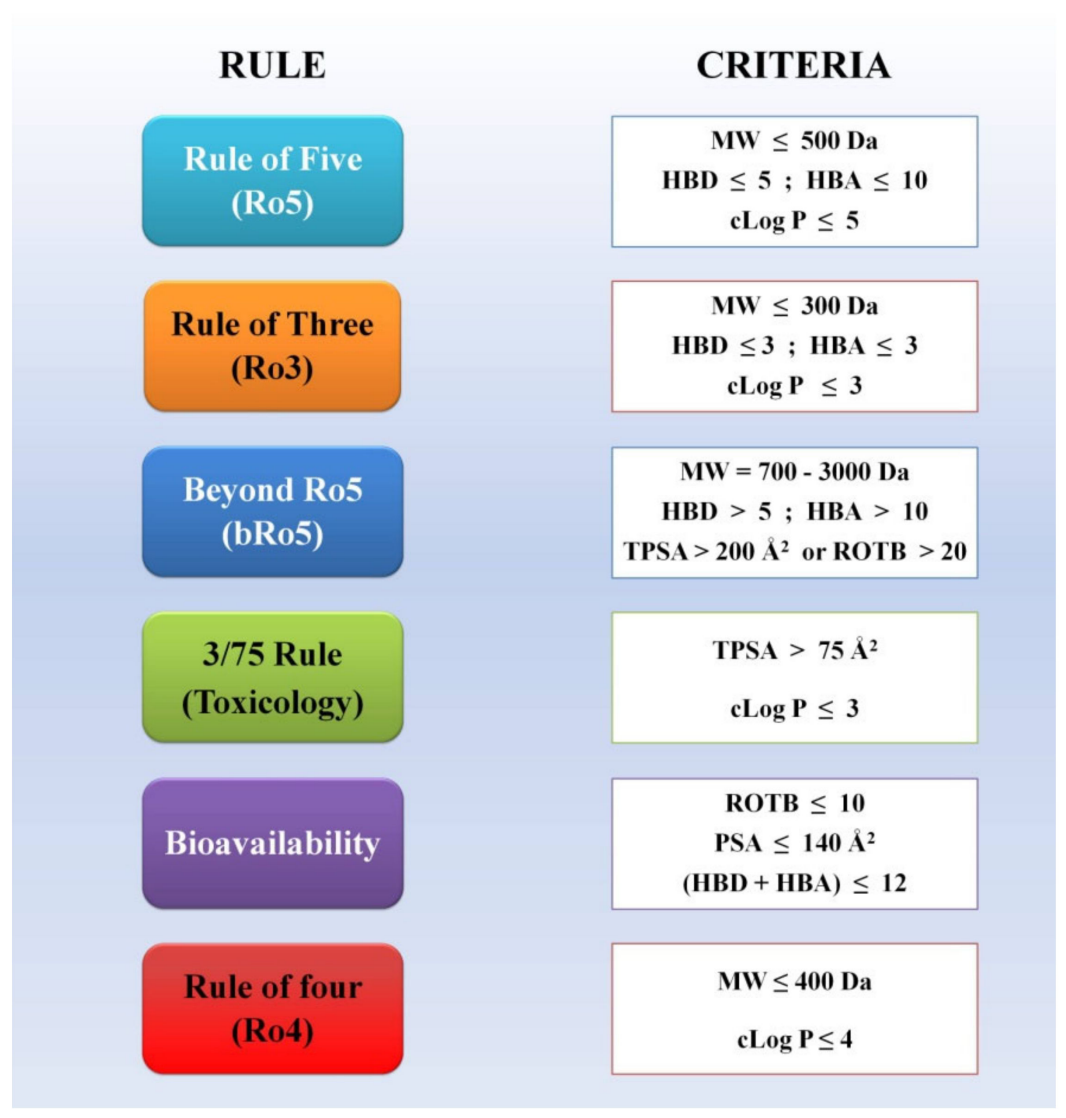
Several commonly applied criteria/rules to identify and characterize the drug-like properties of therapeutic compounds. A comprehensive and extensive list appears in [83] while specific details of each rule are described accordingly: Ro5 [61]; Ro3 [84]; bRo5 [69]; 3/75 [85]; Bioavailability [79]; Ro4 [85].

The optimization process in FBDD arises from a small fragment (e.g., MW~150 Da; millimolar binding affinity) in which most atoms are involved in the desired target–ligand interaction. Therefore, the size, complexity, and physical properties of the molecule are more easily controlled relative to a higher-affinity compound containing groups that are not essential for the desired binding (e.g., MW~400 Da; K_d_~nM). Less potent fragments may undergo high-quality interactions [48] that form the basis for optimization into larger drugs exhibiting enhanced potency [80,81] (refer to Figure 1). Advancement of drug optimization protocols led to the realization that Ro5 and/or bRo5 did not adequately address the needs of FBDD, and therefore, other metrics have been introduced to fill this void. A newly proposed *rule of 3* (Ro3) has been implemented to assist decision making processes involving FBDD [82]. Analogous to Ro5 and based on prior successes, ideal fragments should adhere to the following Ro3: MW < 300 Da; ClogP < 3; HBD < 3; HDA < 3; and, ROTB < 3. FBDD employs fragment-sized compounds that usually comply with the Ro3 for initial screening against a biomolecular target. In this respect, FBDD has better chances of hit identification due to its more efficient sampling of the chemical space. An added advantage is that smaller sized fragment hits are more amenable to structural optimization [82]. Challenges associated with this strategy include the sensitivity of detection methods as discussed in a subsequent section. A significant number of criteria and rules have been proposed as metrics for evaluating prospective compounds in terms of achieving ideal drug-like properties. Representative examples are summarized in Scheme 1 and a comprehensive list is described elsewhere [83].

#### 2.2.2. Ligand Efficiency (LE)

**Figure 5:**
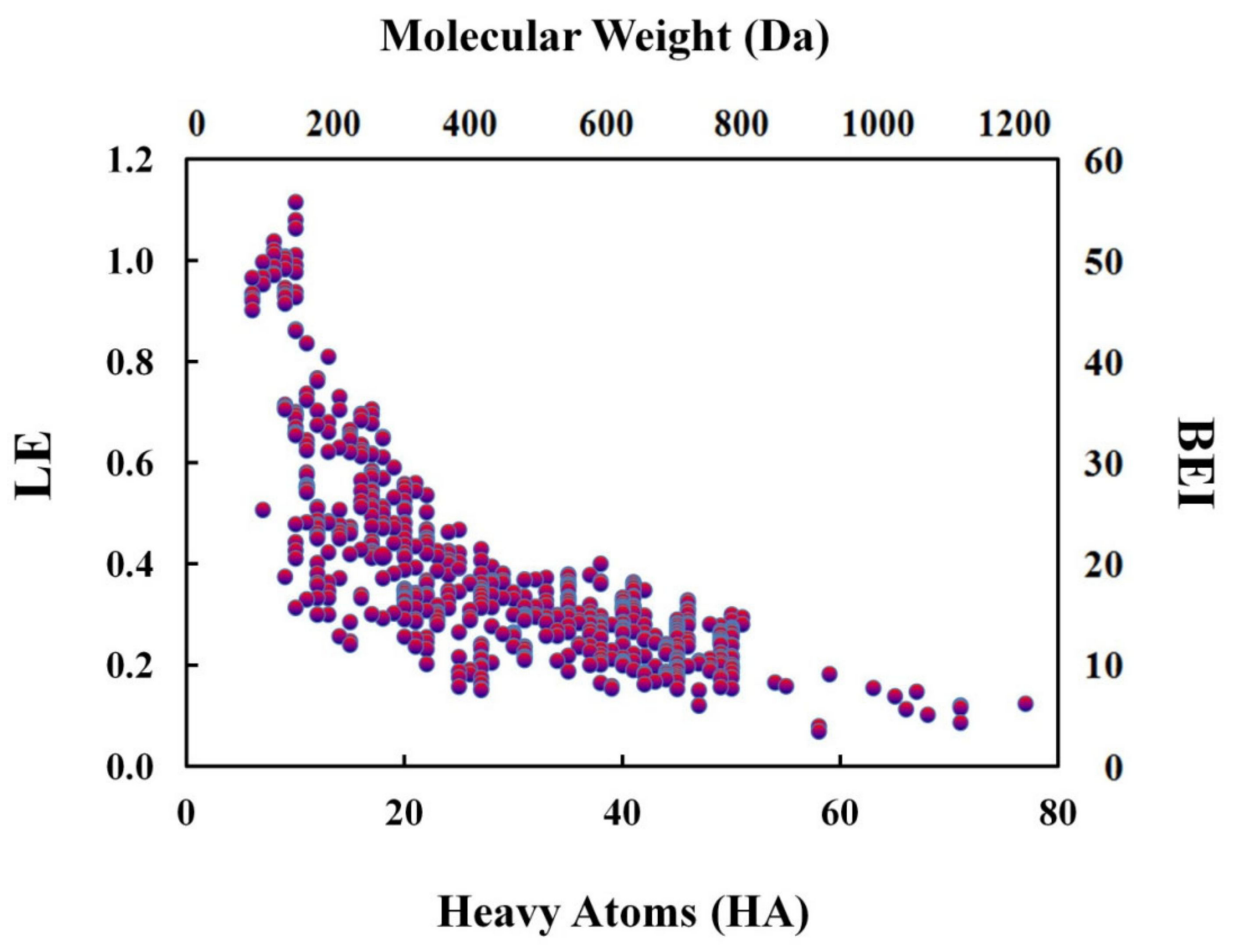
Graphical representation of calculated ligand efficiency (LE) and binding efficiency index (BEI) as a function of Molecular Weight (Da) and Heavy Atoms (HA) [66] curated from public databases [13] and converted as follows: LE = ΔG/HA; BEI = IC_50_/MW (Da) [83,88].

The concept of ligand efficiency (LE) has been proposed [86] in accordance with the observations of Kuntz and colleagues [87] to counter the common misperception that larger molecules generally represent superior compounds. Ligand efficiency is a parameter introduced to avoid the biases of molecular weight differences and normalize the binding affinity on a MW basis [65]. LE is considered a useful indicator of compound quality [83] since the measured binding affinity is normalized to the number of heavy atoms (HA). The popularity of this metric is directly associated with the fact that FBDD has expanded steadily, thereby necessitating emphasis on the optimization of low molecular weight compounds. The binding energy per heavy atom or binding ‘efficiency’ of a ligand has been deemed a useful parameter in the selection and development of a lead compound or fragment irrespective of its molecular weight. The binding efficiency index (BEI) is another metric based on a molecular weight scale [88] that provides a facile and effective ranking en route to compound optimization. The overall utility of these parameters in the drug discovery process are visualized by plotting LE or BEI derived from available binding databases as a function of molecular size. A graphical presentation of the resultant data in Figure 5 reveals hyperbolic behavior with a tendency to reach a plateau at ~25 heavy atoms and MW~400 [66]. Comparing lead compounds on the basis of LE/BEI might prove useful to assess the potential for further optimization of particular ‘hits’ and chemical scaffolds, providing LE is not employed as a *definitive cut-off* in the filtering process. The latter incurs a risk of inadvertently overlooking and potentially discarding useful leads in the drug development process.

A critical and fundamental observation is that these metrics rely on ΔG, K_d_, K_i_, or IC_50_ and thereby render the resultant parameters dependent on a concentration used to define the standard state. Accordingly, a number of investigators have questioned their validity [89,90,91], and several critics have concluded that LE should not be used in absolute terms for individual compounds nor considered as a cut-off criterion [90]. In the midst of this ongoing debate [92] and irrespective of warnings against overusing such metrics as an absolute filter in drug development [91,93], several investigators have adopted a conciliatory approach by reiterating the utility of this metric for specific applications [83,94,95,96]. Alternate metrics such as group efficiency (GE) utilizes differential values (i.e., ΔΔG/ΔHA or ΔpK_d_/ΔHA) to assess the impact of a fragment on compound affinity [93] as described below.

#### 2.2.3. Group Efficiency (GE)

Group efficiency (GE) is a measure of the relative contribution that a specific group confers to ligand potency and is evaluated systematically by differential (ΔΔG) analysis [97,98] involving the comparison of compounds harboring each successive substituent. The latter is related to Free-Wilson relationships [99] that mathematically assess the contribution of group substitutions via QSAR. Considered a more sensitive metric, GE bears the anchoring principle in which ΔΔG refers to the fragment atoms (i.e., ΔHA) that form each succeeding derivative via the following relation: GE = ΔΔG/ΔHA or ΔpK_d_/ΔHA. The interactions between fragment and target should exhibit high ligand efficiency and favorable binding thermodynamics to offset the rotational and translational freedom lost during complex association. Accordingly, fragment binding is generally enthalpy-driven to overcome the loss of rigid-body entropy [57].

**Figure 6:**
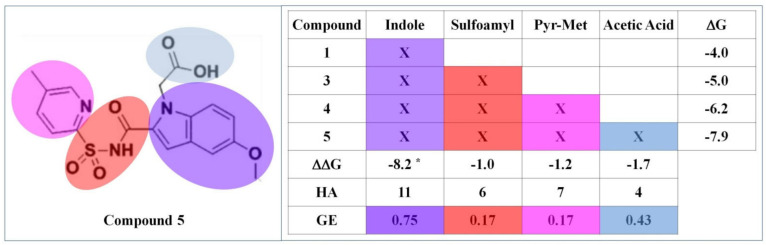
Utility of employing group efficiency (GE) as a metric in lead optimizations [100]. The binding affinity acquired via ITC measurements yields the corresponding free energy (ΔG) for each derivative. The resultant ΔΔG and heavy atom (HA) numbers facilitate calculation of group efficiency (GE) via the relation GE = ΔΔG/HA. Compound **5** is deconstructed into its constituent fragments (i.e., **1** = indol (purple); **3** = **1** + sulfamoyl (red); **4** = **3** + pyridine-methyl (magenta); **5** = **4** + Acetic acid moiety (blue). * The ΔΔG of −8.2 kcal·mol^−1^ includes an initial ΔG_rigid_ (i.e., 4.2 kcal·mol^−1^) that is added to the measured ΔG of −4.0 kcal·mol^−1^.

The evaluation of fragment binding efficiency to overall molecular performance is deduced via the relation: ΔG = ΔG_int_ + ΔG_rigid_, where ΔG_int_ is the intrinsic binding free energy and ΔG_rigid_ is the free energy associated with loss of rigid body entropy upon interaction with the target [97]. The latter has been estimated as 4.2 kcal·mol^−1^ [81,97] and is incorporated in the initial scaffold fragment binding free energy as illustrated in Figure 6. This particular GE analysis systematically evaluates and improves the potency of pantothenate synthetase inhibitors against Mycobacterium tuberculosis [100] as drug candidates in tuberculosis therapy. Inspection of the free energy landscape reveals that compound 5 is dissected into its component fragments and the binding contributions (ΔΔG) calculated from these building blocks. The resultant ΔΔG (−8.2 kcal·mol^−1^) for the indole group (i.e., compound 1) includes an unfavorable entropy loss upon binding [i.e., ΔG = ΔG_int_ (−8.2 kcal·mol^−1^) − ΔG_rigid_ (+ 4.2 kcal·mol^−1^) = −4.0 kcal·mol^−1^]. The addition of successive sulfoamyl, pyridine-methyl, and acetic acid fragments significantly enhances ligand binding free energy. Fragment linking approaches utilizing GE as a metric therefore facilitate the optimization of these compounds, leading to improved inhibitor potency against pantothenate synthetase.

#### 2.2.4. Lipohilic Ligand Efficiency (LLE)

Based on the original hypothesis advanced nearly 35 years ago [101] that advocated for enhancing drug hydrophilicity while preserving overall efficacy, the LLE concept has withstood the test of time as a molecular descriptor in drug design and development [86]. Ligand lipophilic efficiency (LLE) or LipE has received significant attention recently, as this metric combines potency and lipophilicity in such a manner that drug efficiency is normalized to its degree of lipophilicity (as reviewed in [102]). LipE is considered a metric that predicts the quality of a compound and encapsulates both lipophilicity and potency. This parameter is defined according to the relation: LipE (LLE) = pKd (pIC50) − LogP (aLogP, cLogP), where the terms in parentheses are interchangeable with those in the equation. LipE therefore provides an estimate of ligand affinity that is primarily derived from polar interactions as opposed to its hydrophobicity characteristics. In contrast with LE, which has been the subject of significant scrutiny for a number of aforementioned reasons [91,93], LipE is broadly accepted and considered a reliable metric in the overall drug screening and optimization process [102].

**Figure 7:**
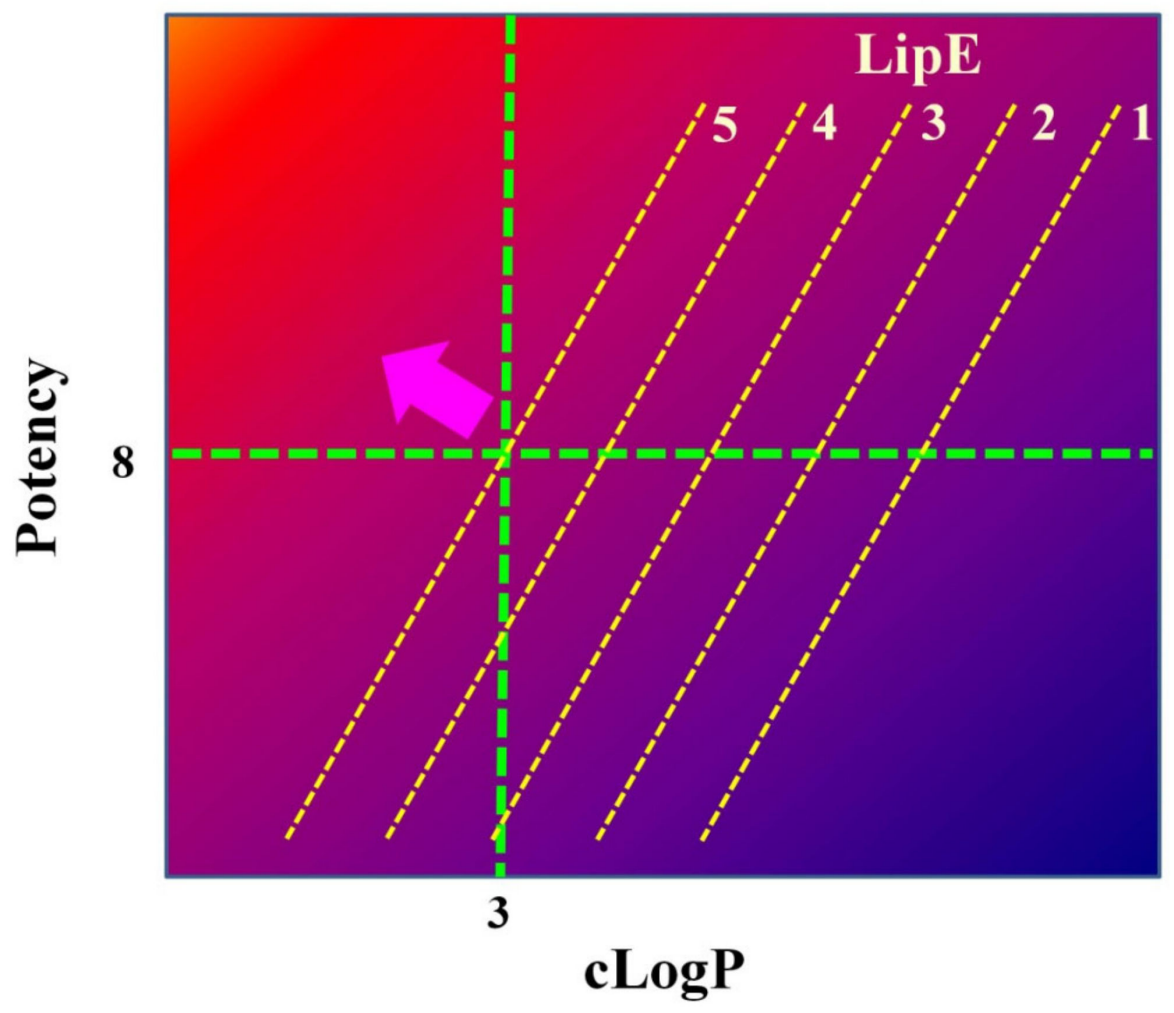
Hypothetical representation of a compound exhibiting a potency (pK_d_) of 8 and octanol- water partition coefficient (cLogP) of 3 resulting in a LipE of 5 (i.e., LipE = pK_d_ − cLogP). The horizontal green dotted line projects outcomes where LogP increases without changes in affinity yielding a significant reduction in lipophilic efficiency. Alternatively, the vertical green dotted line demonstrates how potency increases at constant cLogP resulting in enhanced lipophilic efficiency. A magenta arrow represents the direction for improvement of compound properties.

The utility of estimating LLE (LipE) may be illustrated by the hypothetical representation in Figure 7 that depicts a compound undergoing optimizations to acquire potencies greater than 8 (e.g., 10 nM affinity) yet maintaining low LogP values (e.g., <3) with a consequent enhancement in overall lipophilic efficiency [103]. While the LLE metric represents an excellent criterion to guide compound optimizations, there are competing factors, such as membrane permeability, that must be considered when assessing overall drug quality. This realization infers that there is a limit to which optimizations should focus solely on potency and low LogP, as there is a delicate balance between lipophilicity and solubility to achieve both potency and suitable ADMET properties [104].

#### 2.2.5. Enthalpic Efficiency (EE)

Thermodynamics has been employed extensively to assist decision making processes in drug design and development. Considering the relevance of employing thermodynamic signatures in lead optimizations [6,105,106,107,108,109,110,111,112], the metric of *enthalpic efficiency* (EE) has been proposed as a primary decision criterion in drug discovery [113]. This metric is calculated using the measured binding enthalpy (i.e., ΔH) that is normalized to the number of non-hydrogen (N_NH_) or heavy atoms (HA) (i.e., EE = ΔH/N_NH_) in the molecule. The foundation of this approach is based on the seminal findings published by Freire and colleagues [114], who demonstrated that drug performance may be optimized more effectively via detailed knowledge of the binding thermodynamics. Based on these findings, the energetic signatures of ligand–target interactions represent a core metric for evaluating lead compound quality and accelerating drug design efforts [110,115,116].

Several of the metrics described in previous sections incorporate affinity (K_a_) and/or inhibition (K_i_, IC_50_) data derived from a host of methodologies that are subsequently converted to the binding free energy (ΔG). In contrast, the EE metric requires rigorous determination of complete thermodynamic binding parameters [i.e., free energy (ΔG), enthalpy (ΔH), and entropy (TΔS)] via model-independent analytical techniques, such as isothermal titration calorimetry (ITC). An alternative approach is to employ model-dependent van’t Hoff optical measurements or robust computational methods [117] as substitutes for the more precise calorimetric instrumentation.

**Figure 8:**
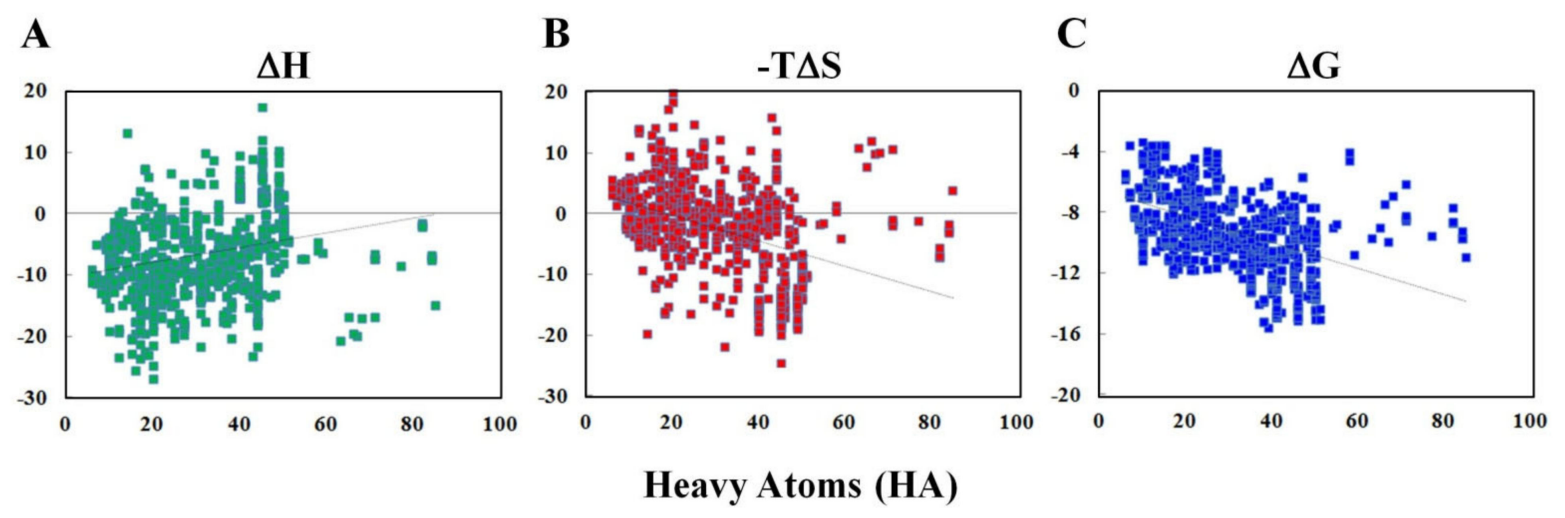
Correlation of ligand-target thermodynamic binding parameters in Panels (**A**) Enthalpy, (**B**) Entropy, and (**C**) Free Energy as a function of Heavy Atom (HA) number curated from public databases [13]. Inspection of the respective plots reveals qualitative trends including a slight reduction in favorable enthalpy and enhancement in both favorable entropy and free energy with increasing number of heavy atoms.

**Figure 9:**
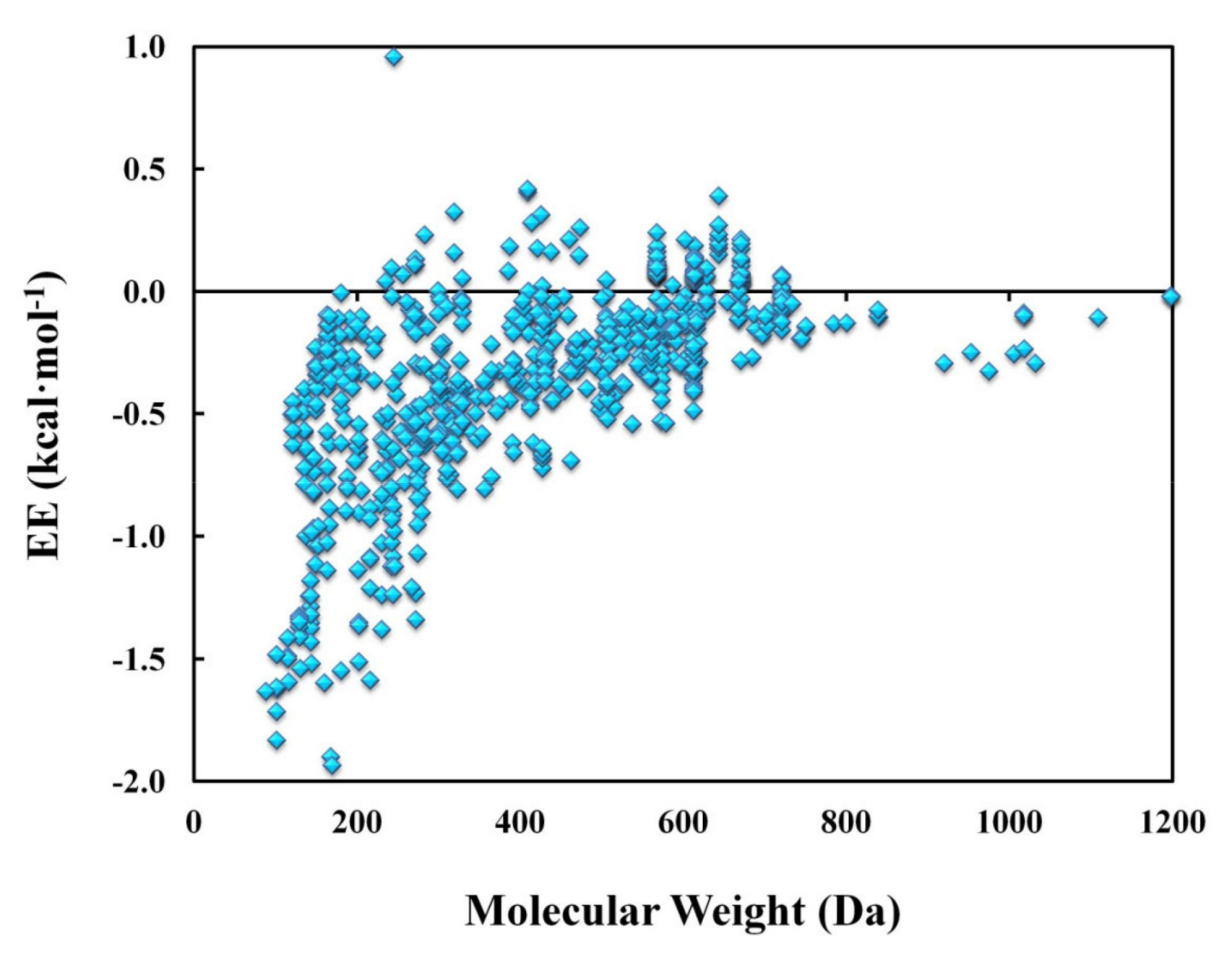
Enthalpic efficiency (EE) calculated using the measured binding enthalpy (ΔH) normalized to number of non-hydrogen (N_NH_) or heavy atoms (HA) (i.e., EE = ΔH/N_NH_). This approach is based on the finding that drug performance may be optimized more effectively via knowledge of binding thermodynamics [114] in which the energetic signatures of ligand-target interactions assist drug design efforts.

The proliferation of thermodynamic binding parameters has facilitated curation of energetic databases that are useful for observing specific correlations and/or trends that may be evaluated to identify commonalities and develop further predictive capabilities. As an example, inspection of published databases reveals a qualitative trend in which the binding enthalpy (ΔH) decreases with MW as illustrated in Figure 8A. Conversely, the entropic (−TΔS) term depicted in Figure 8B exhibits a favorable correlation with increasing size, an observation that is consistent with the burial of larger surface areas upon binding. As a direct consequence, binding-induced apolar surface area desolvation is characterized by a slight increase of ΔG as observed in Figure 8C. While the binding enthalpies tend to gradually decrease as a function of MW, one observes a sharp molecular weight-dependent reduction when the ΔH values are converted to EE, as visualized in Figure 9.

**Figure 10:**
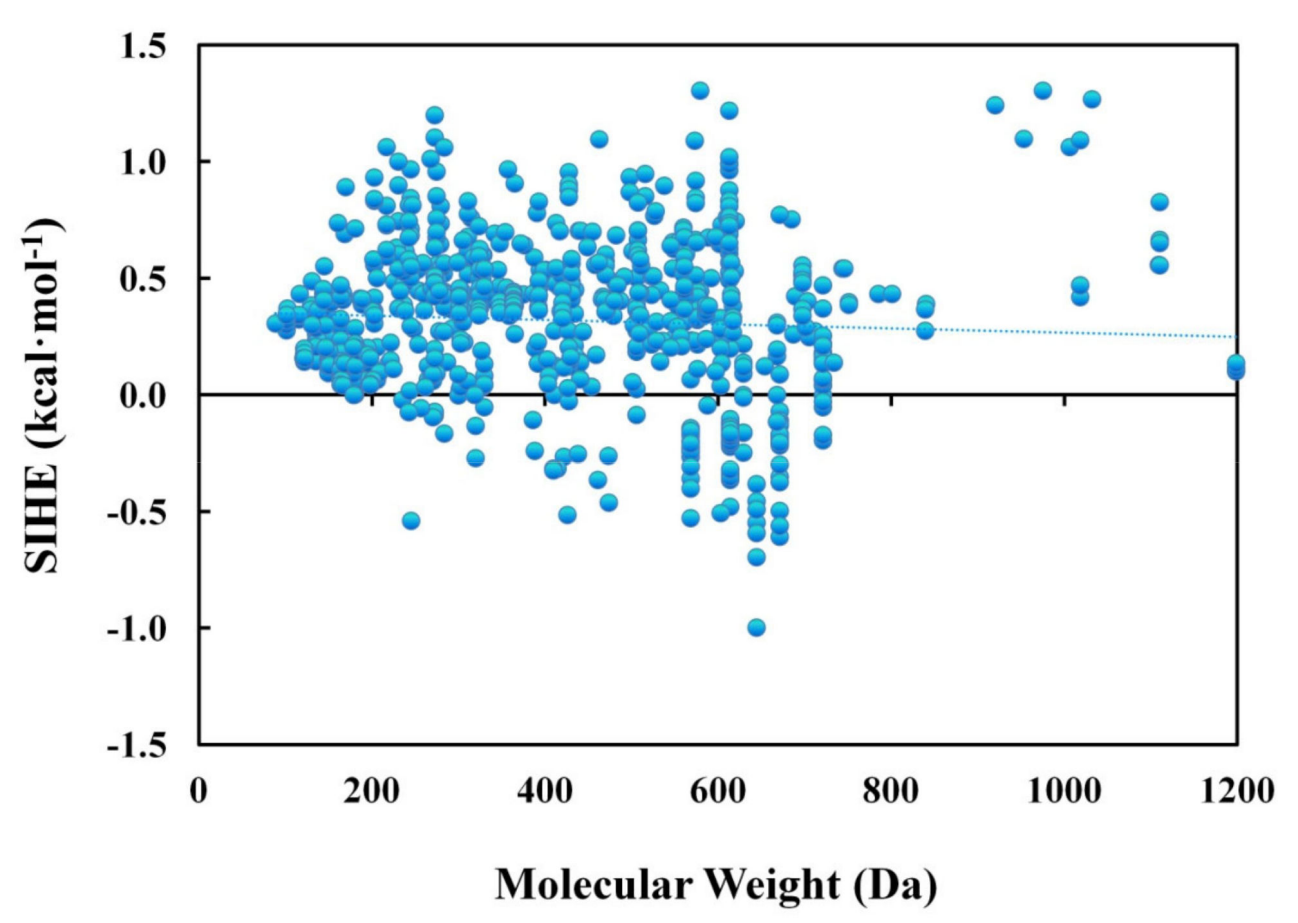
Size-independent enthalpic efficiency (SIHE) defined as p*K*_H_/40 · HA^0.3^ where p*K*_H_ = ΔH/(2.303 · RT) and HA is the number of heavy atoms [13].

Subsequent efforts to guarantee an unbiased hit selection and further hit-to-lead progression have culminated in the development of a *size independent enthalpic efficiency* (SIHE) metric [13], which normalizes EE by the number of heavy atoms as illustrated in Figure 10. SIHE is defined as p*K*_H_/40·HA^0.3^, where p*K*_H_ = ΔH/(2.303·RT) and HA is the number of heavy atoms [13]. This metric has been proposed on the basis of its relevance to monitor enthalpic gains/losses during lead optimization while simultaneously normalizing the data to molecular size. SIHE represents an informative metric for analyzing MW-independent trends in binding enthalpies following normalization for molecular size.

Since binding affinities and inhibition constants may miss important physical properties of a lead compound, complementary approaches that exploit thermodynamic binding properties provide an additional level of critical information which has proven enlightening in the drug discovery process. The compilation of thermodynamic databases has facilitated evaluation of binding energetics as a core metric in lead compound development, resulting in a number of interesting qualitative observations (as reviewed in [13]), namely: (a) drug binding affinities tend to increase with MW; and, (b) enhanced binding free energies are generally accompanied by a reduction in enthalpy and increase in entropy. These findings suggest that EE has a fundamental role in FBDD optimization approaches as the initial screening process should focus on identifying enthalpy-dominated binding fragments [53,118,119] to overcome the natural tendency for a size-dependent reduction in enthalpic efficiency. The early stages of lead development and optimization therefore benefit from a rigorous assessment and characterization of thermodynamic binding signatures.

#### 2.2.6. Exploiting LipE and EE as Core Metrics to Achieve Optimal Success

**Figure 11:**
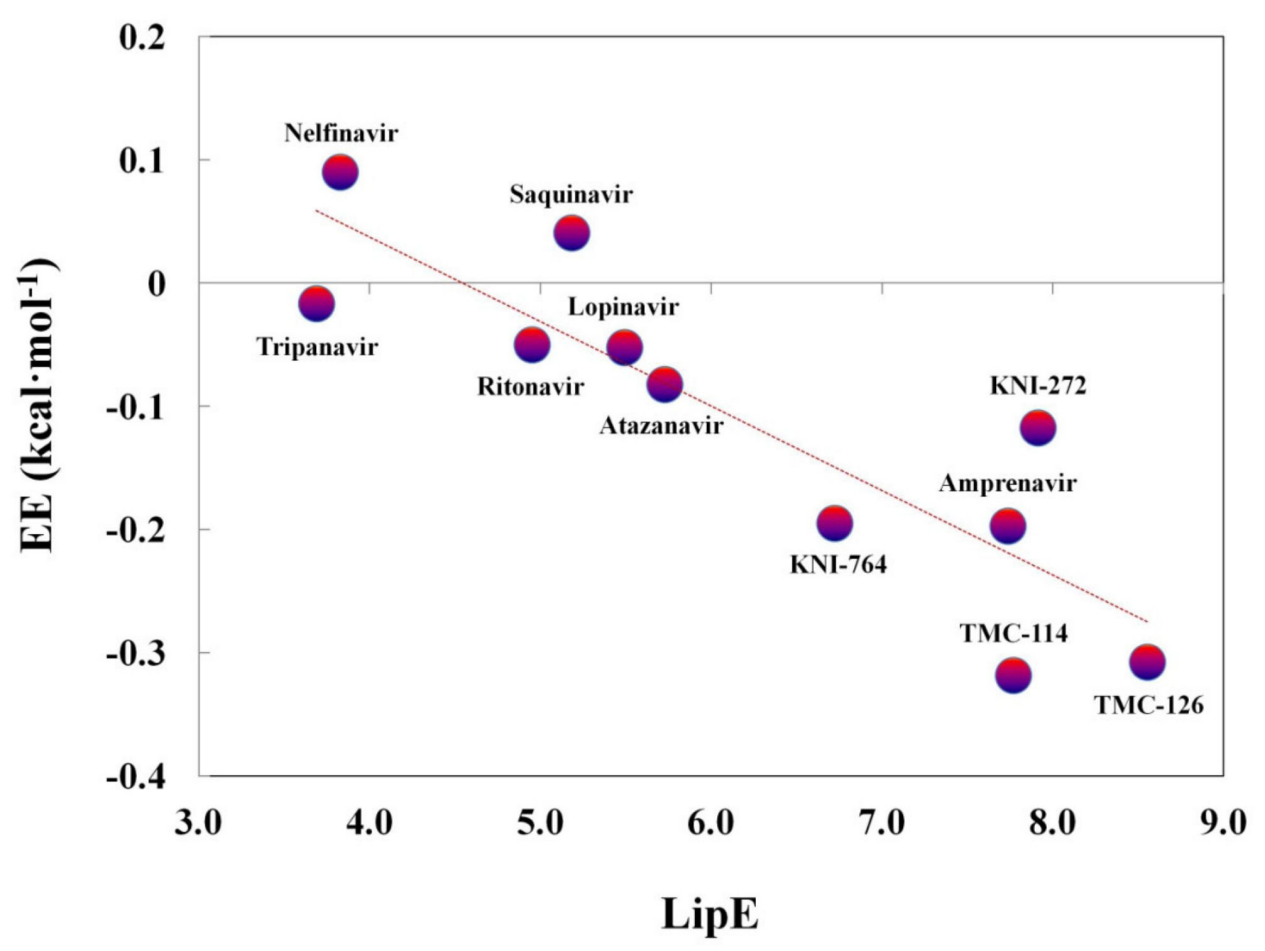
Correlation of enthalpic (EE) and lipophilic (LipE) efficiencies for HIV protease inhibitors retrieved from the PDcal database. The overall distribution reveals that ligands characterized by higher LipE generally exhibit a more favorable enthalpic contribution upon association with the target.

Considering the strengths and weaknesses of specific metrics discussed in the preceding sections, the number of available analytical tools to assist decision making in the drug discovery process allows a robust lead evaluation. The combined efforts of structural, computational, and biophysical methods, in conjunction with desired biological properties, provide assurance that drug discovery protocols achieve the ultimate goal of hitting a specified target. Following two decades of establishing rules and metrics as guidelines in the drug discovery arena, several criteria stand the test of time for evaluating lead compound quality during the early stages of development and subsequent optimization into a successful therapeutic agent. Although the use of a metric does not necessarily guarantee or increase the probability of success, a wealth of evidence suggests that LipE might be viewed as a core metric that best describes compound quality (as reviewed in [67]). Remarkably, considering its emphasis on polarity and lipophilicity control, this metric serves as a gauge of ADMET properties and therefore tracks with the EE of a ligand [120]. An example of their collective utility is depicted in Figure 11 for HIV protease inhibitors whereby one observes a reasonable correlation between LipE and EE. Significantly, both of these metrics track with drug development, thereby corroborating the notion that LipE and EE reflect ligand properties to a similar degree [67].

**Scheme 2:**
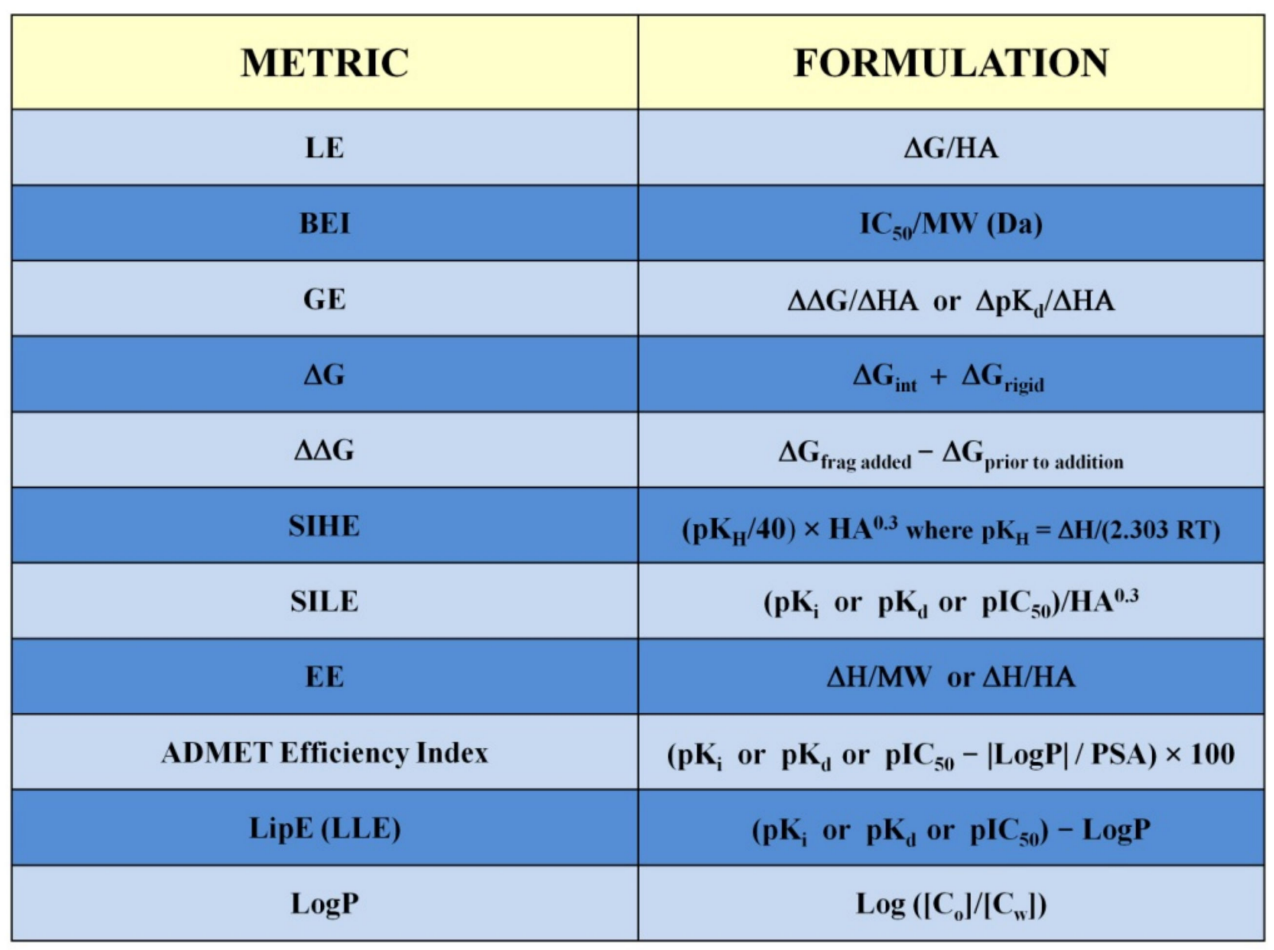
Several commonly employed drug design metrics applied to prioritize hit/lead compounds and their respective formulations. Specific details of each metric are reported elsewhere: LE [65]; BEI [88]; GE and related calculations [98]; SIHE [13]; SILE [121]: EE [113]; ADMET Efficiency Index [122]; LipE (or LLE) [123]; LogP [61].

Enthalpic optimization has proven invaluable at early stages of drug discovery, given its unique power and versatility in discriminating compounds on the basis of selectivity and improved ADMET properties. Collectively, the LipE and EE metrics exhibit reasonable correlation and generally represent efficient criteria for discriminating between selective versus promiscuous ligands. The application of these core metrics at the outset of lead optimization may therefore assist in achieving successful outcomes. A detailed review [120] using available binding databases critically evaluates correlations between LipE and other metric indices, including enthalpic efficiency of ligand–target systems, and documents the advantages and caveats of these approaches. Scheme 2 summarizes the core metrics that are commonly employed in drug design and lead optimization protocols. The reader is referred to a detailed review that contains a comprehensive and extensive listing of drug discovery metrics [83].

#### 2.2.7. Pre-Screening Library Fragments Conserves Resources/Time and Is “PAIN-Less”

Initial fragment libraries contain a myriad of potential therapeutic compounds that must be evaluated in accordance with specific criteria and metrics designed to identify prospective leads. The resultant screening process is resource/time intensive and often compromised by “bad actors” that may ultimately prolong drug discovery protocols [57]. These “misbehaving” molecules may include nonspecific ligands, reactive modifiers, chelators, and/or aggregating compounds that represent undesirable false positive hits [124]. An initial pre-screening inspection of prospective fragments can ascertain whether a specific library contains molecules that are formally included in the PAINS (Pan-Assay Interference Compounds) database [125]. There are a number of recommendations for pre-cleaning fragment libraries prior to initiating compound screenings [57] and several PAINS pre-filters have been proposed as described elsewhere [126]. PAINS filters may assist the drug screening process by eliminating promiscuous and reactive compounds that are “frequent hitters” and unlikely to represent useful leads. Orthogonal approaches are recommended as caution should be exercised whenever implementing cut-off filters to ensure that an otherwise potentially innovative drug candidate bearing possible PAINS alerts is inadvertently overlooked.

## 3. Insights Gleaned from Thermodynamic Binding Signatures

### 3.1. Enthalpy-Entropy Plots

**Figure 12:**
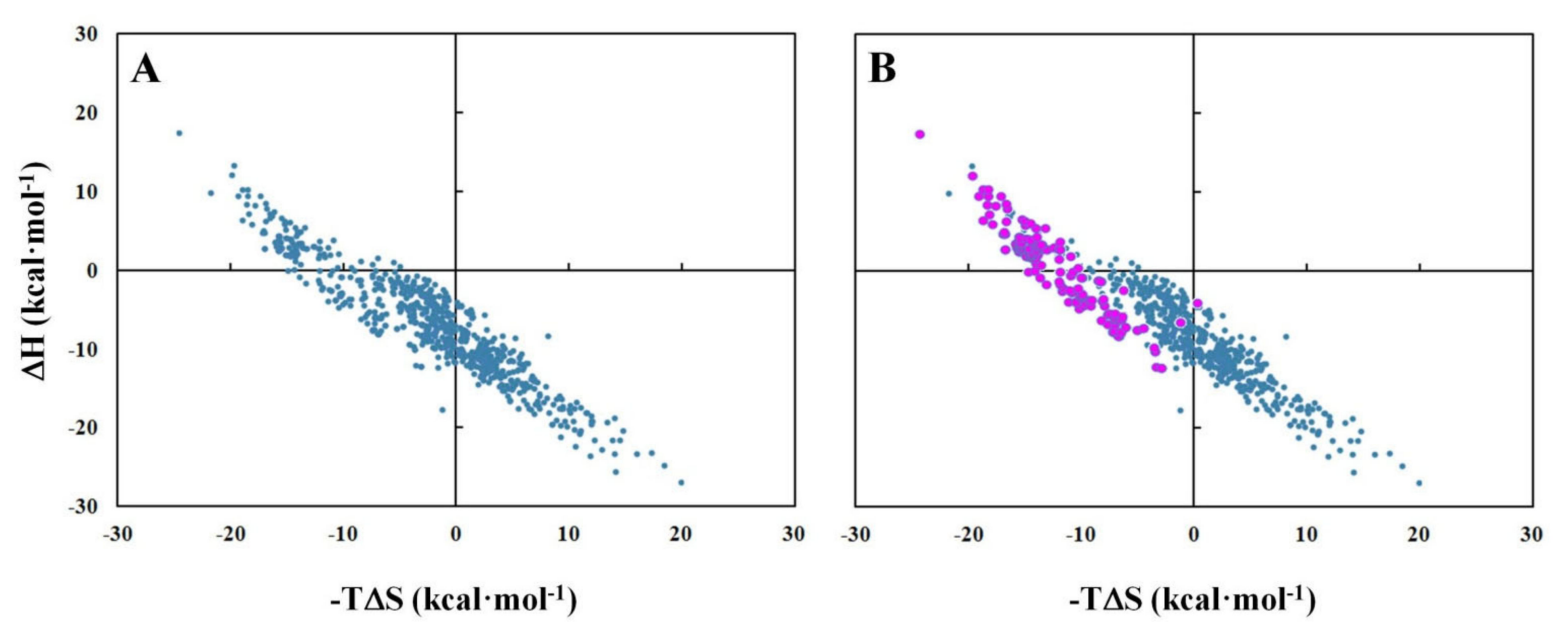
Distribution of compounds retrieved from databases of protein-ligand complexes according to their corresponding enthalpic (ΔH) and entropic (−TΔS) signatures. (**A**) Linear correlation of ΔH and −TΔS reflecting interactions that are enthalpically unfavorable and entropically favorable (top-left quadrant); enthalpically and entropically favorable (bottom-left quadrant); enthalpically favorable and entropically unfavorable (bottom-right quadrant). (**B**) Linear correlation of ΔH and −TΔS highlighting HIV-protease inhibitor interactions as magenta spheres spanning the top- and bottom-left quadrants. The strategy of enthalpic optimization is signified by a gradual shift in distribution of these compounds towards the lower quadrant.

Protein–ligand binding databases provide valuable insights regarding the thermodynamic properties of drug-target interactions. Critical analysis of available binding data furnishes information on the utility of obtaining thermodynamic parameters in drug discovery efforts. An instructive example of this strategy appears in Figure 12A which depicts a subset of binding data cast in the form of ΔH/TΔS linear plots divided into four quadrants. Based on this graphical representation, it is possible to visualize the density of interactions that are either entropy-driven (top left quadrant) or enthalpy-driven (bottom right quadrant), versus those that are both enthalpically and entropically favorable (bottom left quadrant). As proposed by Freire [110], an ideal scenario is to optimize enthalpic contributions to the interaction such that a compound achieves a greater selectivity towards its target, with the added advantage of preferred physical properties vis-à-vis solubility characteristics.

An example of applying enthalpy-entropy plots to track drug development is illustrated in Figure 12B for a series of HIV protease inhibitors against wild type and mutant variants (highlighted in magenta). This representation of the data reveals that binding interactions are distributed within two major groups, namely those that are entropy-driven (ΔH > 0; −TΔS <0) and those that are both enthalpy- and entropy-driven (ΔH < 0; −TΔS < 0). In a retrospective analysis spanning 12 years since the commercialization of these drugs, it is interesting to note that binding data corresponding to the introduction of newer compounds progressively populated the lower quadrant, which comprises enthalpically favorable interactions, as noted in Figure 12B. This finding is consistent with enthalpic optimization [24,109], a strategy that has gained momentum since the original proposition over two decades ago [109,114]. Freire and colleagues [115] have further illuminated the roadblocks to drug optimization (as reviewed in [24]) visualized through the lens of an optimization funnel as discussed below.

### 3.2. The Optimization Funnel

**Figure 13:**
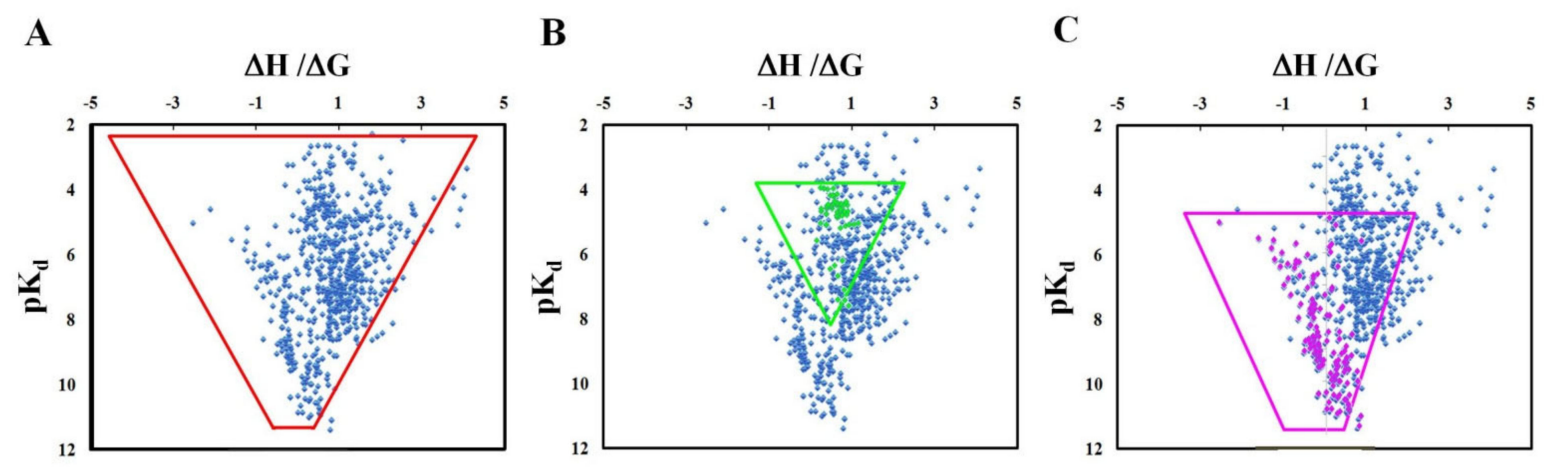
Optimization funnels generated by plotting pK_d_ as a function of ΔH/ΔG ratios for (**A**) protein-ligand interactions (red) derived from thermodynamic databases [13] highlighting (**B**) trypsin inhibitors (green) and (**C**) HIV protease inhibitors (magenta).

In the pursuit of novel therapeutic agents, a common goal is to identify high affinity potent compounds, and the design of optimization filters can represent an informative metric for monitoring the drug development process [115]. An observation gleaned over the years is that the optimization of a chemical scaffold results in high affinity/high potency compounds. Generally speaking, micromolar binding affinities (*K*_d_) are enhanced three to six orders of magnitude (i.e., nano or picomolar) with a corresponding binding free energy increase of ΔΔG ~ 4–7 kcal·mol^−1^ [115]. A practical demonstration of ligand optimization is easily visualized by inspection of the enthalpic (ΔH) contribution to net binding free energy (ΔG) as the affinity of a compound is optimized [24]. In the absence of optimization, low affinity compounds can exhibit a broad range of ΔH/TΔS combinations [115] as a consequence of intrinsic hydrophobic and polar interactions. As the binding affinity increases due to compound optimization, the range of available combinations narrow and converge, resembling a funnel as illustrated in Figure 13A. Significantly, optimization funnels are observed in databases containing a wide range of compounds and unrelated targets as the number of enthalpic-entropic combinations is reduced and the average compound affinity increases. By overlaying specific examples within the global data, one observes sub-funnels, such as that of trypsin inhibitors in Figure 13B (green dots) as well as different generations of HIV protease inhibitors complexed to protease variants in Figure 13C (magenta dots). While the average ΔH/ΔG ratio changes with each system studied, a characteristic and common feature is conversion of higher affinity complexes to a narrower distribution within the optimization funnel.

## 4. Biophysical Methods Employed in Drug Discovery

Valuable information can be gained from the judicious inspection of molecular interactions that document outcomes based on compound hits derived from library screenings. There are a multitude of experimental methodologies and techniques available for initial screenings of fragment libraries [48,53,127]. These include nuclear magnetic resonance (NMR), surface plasmon resonance (SPR), thermal shift assay (TSA), capillary electrophoresis (CE), microscale thermophoresis (MST), biolayer interferometry (BLI), weak affinity chromatography (WAC), grating-coupled interferometry (GCI), cryo-electron microscopy (CEM), photoaffinity probes, fluorescence-based techniques, and isothermal titration calorimetry (ITC). This review specifically focuses on the use of ITC both in terms of its unique advantages and experimental challenges.

### 4.1. Characterization of Ligand-Target Interactions via ITC 

ITC is widely regarded as an essential tool in the repertoire of biophysicists for characterizing the energetics of macromolecular interactions. Since its introduction to the scientific community approximately three decades ago, ITC has gained critical acclaim as the experimental technique of choice for the quantitative assessment of association processes. ITC is routinely applied in drug design and therapeutic strategies for the discovery of lead compounds that target specific macromolecules. In drug discovery protocols, ITC assays are typically performed employing millimolar and micromolar concentrations of lead compounds and targets, respectively. The most significant advantage afforded by ITC is that a single well-designed experiment facilitates the precise determination of the association constant (K_a_), Gibbs free energy (ΔG), enthalpy (ΔH), entropy (ΔS), and stoichiometry (n) of a binding interaction.

The simultaneous assessment of binding affinity (K_a_) and energetic driving forces (ΔH, ΔS) renders ITC an indispensable technique for characterizing ligand-target interactions [6]. A complete thermodynamic binding profile assists in elucidating the interrelationships between ligand affinity and overall biophysical properties. Following data acquisition and analysis via specialized methods and programs [128,129,130], the resultant thermodynamic signatures are employed to evaluate the efficacy of prospective compounds on the basis of binding energetics. In addition to the intrinsic value afforded by another layer of information, ITC-derived thermodynamic signatures have been recognized as an invaluable metric in drug design, development, and optimization given their integral role for assessing enthalpic efficiency (refer to the discussion on EE and SIHE in Section 2.2.5). 

The thermodynamic parameters describing a particular ligand-target association process are a function of specific contributions that can be attributed to molecular interactions and driving forces. Parsing the Gibbs free energy into its enthalpic and entropic components permits identification of favorable/unfavorable interactions and discrimination of primary driving forces. Specifically, the binding enthalpy is comprised of favorable (e.g., hydrogen bonding and van der Waals) and unfavorable (e.g., polar group desolvation) ligand–target interactions. Conversely, the binding entropy includes favorable (e.g., desolvation and release of water molecules to bulk solvent) and unfavorable (e.g., conformational and/or motion restrictions) contributions arising from both the ligand and target (as reviewed in [8]). Acquisition of the requisite thermodynamic binding profiles as a function of temperature affords evaluation of heat capacity changes (ΔC_p_) accompanying the binding process [131,132]. This extra-thermodynamic information is essential for elucidating binding processes that are coupled with desolvation and/or folding. 

### 4.2. Overview of ITC Methodology

**Figure 14:**
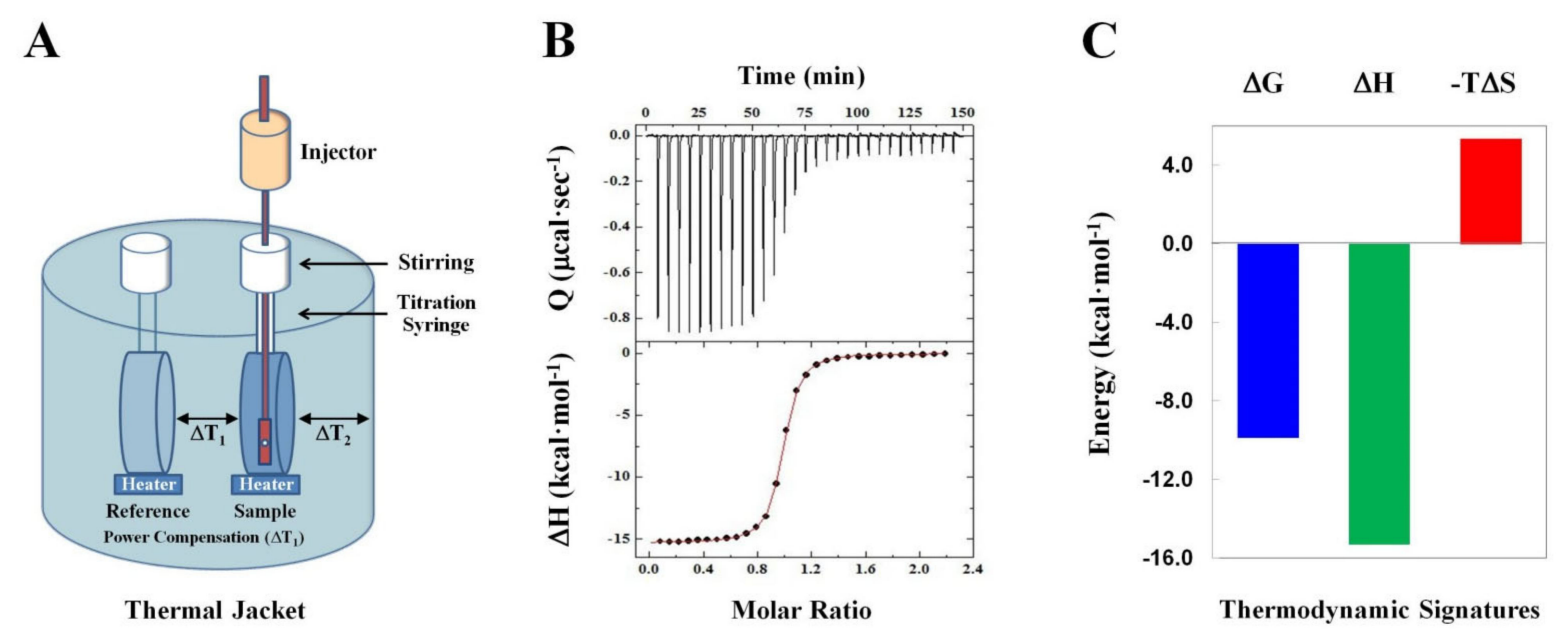
(**A**) ITC schematic depicting basic instrument components housed within an adiabatic jacket that includes a titration syringe delivering precise aliquots of ligand into a sample compartment containing the target solution under constant stirring conditions with the reaction signal detected via thermopiles strategically positioned on the exterior faces of sample and reference cells. (**B**) A representative ITC profile depicting exothermic reaction heats (top panel) integrated and normalized into enthalpy as a function of molar ratio (bottom panel). Non-linear least squares analysis (red line) yields the binding affinity (K_a_), enthalpy (ΔH), and stoichiometry (n). (**C**) Resultant thermodynamic binding signatures derived via analysis of the ITC reaction profile: ΔG (blue), ΔH (green), and −TΔS (red).

The basic principle of operation in an ITC experiment involves the use of a thermostatted titration syringe to dispense precise aliquots of a ligand into the sample cell containing a prospective receptor. The addition of titrant is accompanied by a measurable reaction heat due to ligand dilution and potential interactions with the target. Each ligand injection is characterized by the absorption or evolution of heat, triggering a difference in temperature between the sample and reference cells as illustrated in Figure 14A. The resultant temperature differential causes a feedback system to either lower or raise the thermal power as a means of compensating for this temperature imbalance. The experimental protocol is designed to allow sufficient time between successive injections to restore the temperature balance and thereby ensure that the system achieves equilibrium. 

The thermal signal is detected via thermopiles that are strategically positioned on the exterior faces of the sample and reference cells. An electrical impulse is transmitted to the computer and the heat deflection is registered in the form of an integratable peak per injection, yielding a titration profile that is fit to a binding isotherm (red line) as depicted in Figure 14B. Detailed reviews containing complete method descriptions are available elsewhere [128]. The ITC method is sufficiently robust to accommodate a diverse range of interacting species within biological processes, irrespective of their biochemical and physicochemical properties. ITC analysis affords the evaluation of small molecule interactions with a target macromolecule, as the resultant binding parameters provide an energetic description of the association process in the form of characteristic thermodynamic signatures (i.e., ΔG, ΔH, and TΔS), as illustrated in Figure 14C.

## 5. Thermodynamic Binding Signatures as a Metric of Drug Potency, Selectivity, and Adaptability

### 5.1. Achieving Superior Lead Compound Selectivity

In addition to several lines of evidence that suggest favorable enthalpic interactions optimize compound efficacy, a potential link between thermodynamic signatures and drug selectivity has been proposed [115] and reviewed extensively [133]. These findings infer that improved shape complementarity is directly correlated with enthalpically-driven interactions, which create a bias towards specific targets versus off-targets, thereby enhancing overall selectivity [115]. As a case in point, lead compounds targeting distinct aldose-reductases exhibit high selectivity towards a particular species with the interaction accompanied by a significant enthalpic advantage [134]. In searching for compounds that selectively interact with and inhibit a specific aldose reductase, small molecules harboring a common 3-benzyluracil-1-acetic acid scaffold containing a chloronitrobenzyl group substituent selectively inhibit an aldose reductase (AR), which is implicated in diabetes. In contrast, a compound containing bulkier ortho/meta substitutions targets the cognate enzyme AKR1B10, an aldose reductase associated with cancer. The latter occurs conceivably via displacement of disordered water that is trapped in the enzyme hydrophobic pocket. As a consequence, each of the enzyme–inhibitor complexes exhibits optimal selectivity and their interactions are characterized by greater enthalpic contributions relative to the remaining ligands. These differential preferences afforded selectivity in cell cultures as the first inhibitor can potentially prevent sorbitol accumulation in retinal cells, whereas the second blocks the proliferation of cancer cells.

### 5.2. Achieving In Vivo Efficacy

While binding affinities toward a target tend to correlate with the desired biological property of a ligand, there are cases in which such correspondence is not readily apparent. In fact, there are examples documenting the utility of acquiring thermodynamic binding signatures in addition to seeking improved efficiency, selectivity, and ADMET properties. A study on CD4/gp120 inhibitors [116] has reported correspondence between HIV-1 cell infectivity inhibition and ΔH/TΔS ratios for various compounds, despite modest differences in the overall binding free energies. The rationale for these results resides in the realization that unwanted conformational effects contribute to modulate the ΔH/TΔS balance, the latter serving as a reporter in the selection of compounds that do not elicit such undesired effects. Therefore, thermodynamic optimization has proven of significant value for the selection of compounds that form complexes with the target protein in a “pre-organized” conformation, a finding that can be confirmed via thermodynamic signatures.

In cases where structural information is neither available nor sufficiently reliable to determine whether conformational changes might impact an outcome for a series of inhibitors, the ΔH/TΔS balance may serve as an indicator/predictor of structural alterations. It is interesting to evaluate the applicability of such a hypothesis on a selected class of peptidylnitrile compounds against *T. cruzi* strains [135] for which cruzain binding thermodynamics have been derived [136]. Despite the lack of correlation between cruzain affinity/inhibition and trypanocidal activity, the binding enthalpies (and corresponding enthalpic efficiencies) vary linearly with the PEC_50_ values (r^2^ ~ 0.98) for a series of compounds studied. These findings suggest that a certain degree of conformational constraint is predicted based on the ΔH/TΔS balance measured for this congeneric series of compounds, whereby comparable affinity ligands may exhibit conformational preferences leading to a differential outcome in situ. 

### 5.3. Achieving Adaptability to Drug Resistance Mutations

In general, engineering effective therapeutic agents harboring potent antiviral properties requires identification of specific factors that dictate selectivity and minimize susceptibility to mutations via adaptability (as reviewed in [8]). In drug discovery strategies, an ideal molecule is evaluated in terms of its potency, selectivity, specificity, selectivity, and adaptability to mutations, thereby preventing drug resistance [137]. Collectively, these characteristic properties can be achieved via knowledge of the enthalpic and entropic contributions to inhibitor-target interactions. In fact, such lead optimizations require an inhibitor to maintain interactions with conserved residues that do not normally undergo mutations, while simultaneously acquiring some flexibility to allow interactions with less stable, variable regions in the target that commonly undergo mutations. Although the design of adaptive ligands may incur an enthalpic penalty as a consequence of suboptimal complementarity, such interactions retain an enthalpic character with compensating favorable entropy, thereby maintaining the desired binding affinity to variant targets and exhibiting effective anti-viral activity.

## 6. ITC in Drug Discovery, Development, and Optimization

### 6.1. ITC in FBDD: Case Studies

During the past decade, several reports have documented the use of ITC as a strategic approach in the initial hit-to-lead phases of fragment-based drug design. There is ample evidence to suggest that selection of fragments on the basis of enthalpic efficiency irrespective of binding affinity leads to superior molecules [118]. As a validation technique in drug development and optimization, ITC has proven invaluable as it furnishes novel information on lead compounds for which thermodynamic properties have not been assessed from the outset. Specific examples on the use of ITC in fragment optimization include the identification of ligands for acetylcholine-binding protein (AChBP) [138] and the characterization of small molecule carriers for siRNA [139]. ITC has been utilized in the validation of fragment hits derived from screenings of ligands targeting the anti-apoptotic protein target Bcl-x(L) via automated mass spectrometry [140]. In a subsequent study, investigators employed ITC to evaluate the binding of compounds generated during FBDD campaigns against two functionally distinct proteins, the X-linked inhibitor of apoptosis protein (XIAP) and cyclin-dependent kinase 2 (CDK2) [141]. 

Alternate drug discovery strategies have been proposed including one developed in an academic setting yet suitable for industrial scale applications [142]. This protocol consists of a fragment screening cascade to identify hits employing a combination of differential scanning fluorimetry (DSF), validation by NMR spectroscopy, and final characterization of binding fragments via ITC and X-ray crystallography. Along these lines, ITC has been utilized in the hit validation of halogen-enriched fragments that exhibit low micromolar affinities and high ligand efficiencies [143]. FBDD strategies have been employed to identify suitable antibiotic candidates targeting tRNA (m1G37) methyltransferase (TrmD) from *Mycobacterium abscessus* (Mab) [144], a rapidly growing multidrug resistant mycobacteria. The screening for compound prioritization involved DSF followed by ITC validation, yielding a lead compound comprised of two merged fragments bound to the active site.

In a quest to identify therapeutic agents against the opportunistic pathogen *Pseudomonas aeruginosa,* investigators recently designed and optimized a series of compounds to interact with pseudomonas quinolone signal receptor (PqsR) [145], a key transcription factor that controls bacterial pathogenicity. Hit optimizations monitored via SPR and ITC evaluated the corresponding enthalpic efficiency (EE) of introducing flexible in lieu of rigid linkers in these compounds. While apparently counterintuitive, the finding that flexible linkers boost PqsR activity and enhance anti-virulence potency can be rationalized on the basis of their respective thermodynamic binding profiles [145]. 

### 6.2. ITC in FBDD: Experimental Challenges

Upon combining the selected fragments discovered via FBDD approaches, the ultimate objective is to derive a lead compound with high ligand efficiency. Pursuing this strategy, the initial fragments are expected to exhibit affinities spanning the range of 100 μM–10 mM to generate a final product with affinities on the order of 10 nM [48]. Specialized analytical techniques are therefore required to screen the low affinity fragments. Biophysical methods commonly employed in the fragment screening process include NMR spectroscopy, X-ray diffraction analysis, mass spectrometry, surface plasmon resonance, fluorescence-based techniques, and isothermal titration calorimetry. Since FBDD requires detection of low-potency hits, a caveat on the use of most biophysical techniques is to obtain sufficient amounts of purified protein target (>10 mg) and fragments that are soluble at the concentrations needed (mM) for screening and optimization. Standard operating practice is to acquire ITC measurements on systems that exhibit a moderate range of affinities (i.e., 10^3^ > K_a_ > 10^7^ M^−1^) with target solution concentrations at least tenfold greater than the ligand dissociation constant (K_d_). Despite these recommended guidelines, a well-designed ITC experiment may yield informative results with significantly less material and/or lower affinities.

While the thermodynamic data acquired via ITC is invaluable in FBDD, the tendency is to employ this technique later in the drug development process for compound optimization given its lower sample throughput and higher target protein requirements relative to other biophysical techniques. Considering these experimental caveats, there are success stories in which ITC has assumed a lead role in the entire drug discovery process. Under specific conditions, ITC has furnished valuable information in FBDD decision making processes [138,139,140,142,145,146,147,148]. Although ITC might pose an experimental challenge in initial FBDD screenings, this technique represents a powerful method during the secondary screening stage and might retrospectively provide important clues that assist further improvements and optimization of the starting fragment candidates. Given the material and time constraints associated with ITC characterization of ligand–target interactions, continuing efforts are devoted towards enhancing overall sample throughput and sensitivity. Recent studies have focused on overcoming such experimental limitations by developing enthalpy screening methods with the goal of accelerating data acquisition [149,150] and expanding the high-throughput capabilities of calorimetric instrumentation. 

### 6.3. Protein-Protein Interactions (PPI) as Targets in Drug Discovery 

Drug discovery programs focusing on protein-protein interaction (PPI) inhibitors are challenging and relatively few FBDD approaches have tackled these projects to identify active small molecules against a number of chemotherapeutic targets (as reviewed in [151]). Considering the fundamental role of PPIs in disease, conventional wisdom has postulated that these interactions might be ‘undruggable’, thereby rendering drug discovery efforts targeting these macromolecular systems particularly cumbersome [152]. Recent developments have challenged this notion, as the physicochemical properties of small-molecule PPI modulators undergoing clinical trial progressively demonstrate typical characteristics of drug-like molecules. These findings suggest that future drug discovery campaigns aimed at targeting PPIs may follow traditional design parameters, albeit along the lines of bRo5 space described in Section 2.2.

One successful example of a PPI inhibition by small molecules involves interactions between tumor suppressor BRCA2 and recombination enzyme RAD51 [153]. In this case, fragment hits capable of interacting with the PPI interface have been validated by ITC, NMR, and X-ray approaches. Another relevant approach in drug design is to identify PPI inhibitors that are essential for a particular cellular function/dysfunction and responsible for a number of pathological conditions. In order to develop treatments for oxidative stress-related diseases, a recent study [154] focused on inhibiting the interaction of transcription factor Nrf2 with its negative regulator Keap1, thereby upregulating Nrf2 transcriptional activity. Seeking to identify inhibitors of these PPI interactions, a number of lead compounds inspired by a natural molecule that interacts with Keap1 have been developed and evaluated via multiple approaches including detailed structural and thermodynamic analysis.

**Figure 15:**
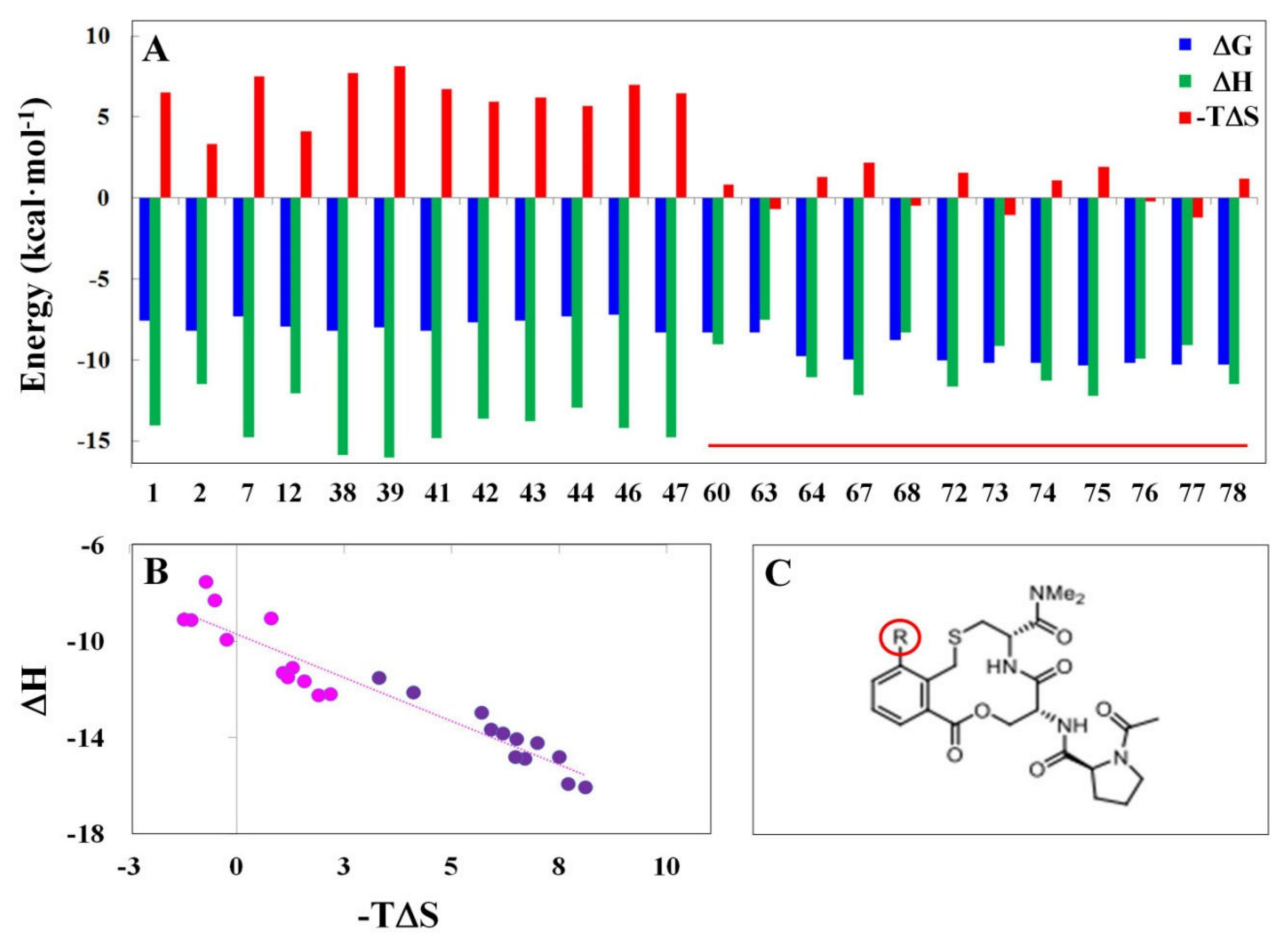
Impact of binding-induced desolvation on the energetic signatures of PPI inhibitors. (**A**) Selected compounds reported in [154] are sorted according to original ID number (**1**–**78**) and comprise two distinct ligand classes on the basis of their respective thermodynamic signatures. Inhibitors **1**–**47** exhibit a higher entropy penalty (red histograms) relative to compounds **60**–**78**, (underlined by a **solid red line**) that are characterized by enhanced affinity as reflected in the higher binding free energy (blue histograms) due to reduced entropic penalty despite the lower binding enthalpy (green histograms). (**B**) An enthalpy-entropy plot discriminates between higher affinity compounds exhibiting lower unfavorable entropy (magenta circles) versus lower affinity compounds (purple circles) characterized by higher unfavorable binding entropies (−TΔS ≥ 3 kcal·mol^−1^). (**C**) Structure of compounds with a substituent R in the ortho-position that exhibit improved binding energetics. Inhibitors **67**, **68**, and **72**−**78** contain either a carboxyl or tetrazole group on an aromatic moiety attached at the ortho-position of the phenylene ring. The entropy gain observed for compounds **60**–**78** may arise from dislodging a coordinated water molecule that bridges residues S508 and R415 of Keap1.

This combined structural-energetic strategy led to improvement of the compound series by introducing chemical modifications that impact solvation and residue flexibility. One of the binding modes involved displacement of a coordinated water molecule, which resulted in an additional entropic gain, complementing the favorable enthalpic contributions of productive key residue interactions. This series of compounds is clearly distinct in terms of respective energetic signatures, as manifest in a striking reduction of the entropic penalty incurred by binding-induced desolvation of the interaction site, as visualized in Figure 15A where entropically favored compounds are designated with a red line (i.e., entries **60**–**78**). The accompanying ΔH/TΔS plot in Figure 15B reveals that these compounds (magenta dots) are clustered in the region of lower −TΔS values (−2.5 < −TΔS < 2.5 kcal·mol^−1^) and variable levels of favorable enthalpic contributions (−12 < ΔH < −7 kcal·mol^−1^). Significantly, the broad variation in thermodynamic signatures occurs via a simple substitution in the ligand as depicted in Figure 15C that conceivably dislodges a coordinated water molecule bridging residues S508 and R415 of Keap1.

Along the lines of modulating PPI interactions as therapeutic interventions, strategies have been developed with the assistance of computational approaches, affording the design of enzyme-inhibiting peptides that transiently convert an active enzyme into a proenzyme. The latter is co-delivered with a pro-drug substrate to specific target sites for subsequent activation by local proteases [155]. This approach eliminates the risks of systemic toxicity as the pro-drug is harmless to the body in its latent form and only converted to an active toxic agent by the accompanying pro-enzyme in local target tissues. Applying this strategy to the therapeutically relevant protein carboxypeptidase G2 (CPG2), a combination of biochemical, computational, structural, and thermodynamic methods has assisted in identifying optimal peptide candidates to fulfill the role of a transient enzyme inhibitor.

### 6.4. Emerging Infectious Diseases: SARS-CoV-2 Therapeutic Interventions 

The emergence of highly infectious diseases such as the SARS-CoV-2 pandemic represents a global health threat that requires development of effective therapeutic treatment regimens. While efforts have intensified to immunize the world population through administration of vaccines, SARS-CoV-2 likely represents a long-lasting endemic that will continue to evolve with the appearance of novel variants evading immunization protocols. In an effort to address this void, therapeutic interventions are urgently needed and recent developments towards this goal have resulted in the approval of several drugs and anti-inflammatory agents. Despite limited progress on this front, specific targets of viral or host origins have been identified for further exploration in the development of prospective therapeutic agents. Current drug discovery strategies have employed a diverse array of biophysical approaches including ITC to assist in validating hits from HTS and characterizing the efficacy of new and repurposed drugs as antiviral therapeutics against SARS-CoV-2 targets.

The coronavirus designated SARS-CoV-2 encodes two proteases, namely 3CL^pro^ (or M^pro^) and PL^pro^, both of which are considered as primary antiviral research targets in SBDD and drug repurposing strategies (as reviewed in [156]). CL-Pro is a class of proteases for which a host of promising molecules have been developed against other coronaviruses. A representative example of these therapeutic agents is GC376 that has already been used to treat feline coronavirus and subsequently evaluated against several SARS-CoV variants. In a recent study, the efficacy of this compound has been validated by ITC and proven specific against SARS-CoV-2 M^Pro^ [157]. A separate study reported on the design and synthesis of dipeptidyl inhibitors with novel P_3_ scaffolds exhibiting potent inhibitory activity against SARS-CoV 3CL^pro^ and nanomolar affinities measured via ITC [158]. The SARS-CoV-2 target PL^pro^ is a papain-like protease with deubiquitinating and deISGylating activities [156]. By removing the ubiquitin-like ISG15 (interferon-stimulated gene 15 protein) modifications from host proteins, this viral protease causes suppression of the innate immune response and promotes viral replication. PL^pro^ is effectively inhibited by GRL0617, a non-covalent inhibitor that interacts with and blocks the association of PL^pro^ with ISG15 [159]. As a consequence, this small molecule inhibits viral replication while potentially promoting anti-viral immunity [160].

Several of the SARS-CoV viral nonstructural proteins including nsp12 (i.e., RdRp) and nsp7-8 (i.e., auxiliary proteins) assemble to form an active replication transcription complex. Employing a combination of biochemical and biophysical approaches, investigators are currently evaluating a number of repurposed compounds against RdRp. Inspired by studies conducted on protein synthesis inhibitors over five decades ago [161], these lead compounds have demonstrated the ability to interact with SARS-CoV-2 RdRp and inhibit ribosomal protein synthesis [47]. The screening and characterization of prospective SARS-CoV-2 therapeutics have been documented including a HTS using the nsp10-nsp16 complex. Repurposed drugs have been assessed for emergency use to treat SARS-CoV-2 infection [162] and the positive hits validated by ITC.

One of the mechanisms underlying SARS-CoV-2 mediated suppression of interferon responses occurs due to orf9b interactions with host cell components. This includes the Hsp90/TOM70 complex for which interactions have been explored structurally and thermodynamically [163,164]. A recent study employing ITC reported on the allosteric inhibition of macromolecular interactions by orf9b [165]. Remarkably, the binding affinity of Hsp90 EEVD motif to TOM70 NTD is reduced by ~29-fold when orf9b occupies the TOM70 CTD pocket, supporting the proposition that orf9b allosterically inhibits Hsp90/TOM70 interactions. This finding sheds light on the mechanism underlying SARS-CoV-2 orf9b mediated suppression of interferon responses [166], thereby providing opportunities for therapeutic interventions.

Another potential SARS-CoV-2 target that has recently received attention is the DC-SIGN (dendritic cell-specific intercellular adhesion molecule 3 grabbing nonintegrin), a C-type lectin receptor that mediates infection and dissemination of numerous viruses [167]. Since the SARS-CoV-2 spike protein is heavily glycosylated, its interaction with DC-SIGN represents a potential ACE2-independent infection route of innate immune cells. Inhibition of virus binding to DC-SIGN therefore represents an attractive host-directed strategy to attenuate overshooting innate immune responses and prevent disease progression. In a recent study, ligand optimization has been monitored via biophysical approaches and calorimetric determination of the resultant thermodynamic signatures for DC-SIGN–glycopolymer interactions revealed a correlation of potency with ligand binding enthalpies [168]. 

An alternate strategy has been pursued to identify aptamers that efficiently bind DNA-susceptible peptide structures in SARS-CoV-2 proteins critical for infectivity such as the receptor binding domain (RBD) of spike protein and SARS-CoV-2 RdRp. By repurposing existing aptamers, investigators have identified positive hits validated by a number of techniques including ITC [169]. The number of studies documenting new anti SARS-CoV-2 candidates published on a daily basis suggest that the use of existing/repurposed drugs, natural chemicals, and/or novel entities has progressively increased since the pandemic onset in 2019. Multiple targets of viral or host origin have already been selected for treatment, as drug repurposing offers an attractive prospect in terms of accelerated therapeutic development. In retrospect, significant progress has been achieved during an abbreviated timeframe given the identification of protease inhibitors and selection of prospective candidates undergoing clinical trials to evaluate their antiviral efficacy against SARS-CoV-2. The arsenal of potential leads and repurposed drugs has advanced dramatically [170,171,172] with the promise of developing more effective SARS-CoV-2 therapeutics to counter an ever-expanding array of novel viral variants.

## 7. Parsing Thermodynamic Binding Signatures

### 7.1. Role of Solvation on Binding Energetics

Numerous studies have succeeded in elucidating the forces driving protein–ligand interactions with particular emphasis on the role of hydration, which is often obscured during static structural analysis or overlooked via computational techniques that do not implicitly or explicitly incorporate solvation models (as reviewed in [173]. The power of computational methods is enhanced by rigorous evaluation of theoretical predictions based on experimental data. In fact, armed with experimental thermochemical and thermophysical data (e.g., ThermoML) [174], molecular simulations can predict specific physicochemical properties of compounds such as infinite dilution activity coefficients (IDAC), which essentially reflect interactions between a single solute molecule surrounded by solvent and may therefore provide valuable insights regarding the solvation energies of compounds [175]. The powerful combination of structural [153,176,177], computational [178,179], and experimental biophysical methods, including calorimetric techniques [180], can furnish invaluable information on the molecular forces driving ligand–target interactions, including the role of solvation in binding energetics.

In principle, ITC measurements yield global thermodynamic signatures that cannot resolve the contributions of each water molecule participating or displaced upon protein–ligand complex formation, and sometimes presents a challenge in overall data interpretation [181]. Examples include certain thrombin–ligand complexes that exhibit nearly identical binding enthalpies despite distinct HB patterns and water networks. This is presumably due to associated structural changes within the interaction site that mask differential binding modes of these compounds [182]. The role of water molecules in ligand recognition is multifactorial, a primary reason why predictions of binding energetics represent a formidable challenge even when structural and thermodynamic information is available [183]. A well-designed multiparametric strategy incorporating systematic group substitutions in the ligand [184,185] and/or amino acid replacements in the target protein [186] may resolve hydration contributions to a ligand-target interaction at the molecular level.

In conjunction with this approach, the acquisition of ITC measurements under rigorous experimental conditions should provide clarification on the origins of net thermodynamic binding signatures. Examples include the use of multiple buffers with distinct ionization enthalpies at different pH to determine intrinsic ligand-target binding enthalpies [187,188,189], and multiple assay temperatures to assess heat capacity changes [132,190]. Some molecular binding events that are detectable at the enthalpy/entropy level are not necessarily reflected in the binding free energy due to enthalpy/entropy compensations as illustrated by oligosaccharide interactions with a number of mutant proteins [186]. The energetics of protein–ligand complexes may be modulated by introducing specific functional groups resulting in enhancement of either the ligand binding enthalpy or entropy. As an example, target–ligand complexes can be stabilized by inserting H-bonding functional groups that interact with or replace interfacial water molecules resulting in a favorable contribution to binding enthalpy. Conversely, the introduction of certain functional groups within a ligand may promote expulsion of surface waters into bulk solvent thereby increasing binding entropy.

The relevance of solvation in molecular recognition events is evident based on numerous observations reported over the years [46,183,191,192,193,194,195,196,197,198,199]. The critical role of solvation is clearly illustrated within the context of drug design and development by citing a classic example of the serine protease family, which includes a number of disease-associated enzymes that have been used as targets in drug discovery campaigns. Klebe and colleagues [183] have provided an illustrative example of individual water molecules eliciting dramatic impacts on the thermodynamic binding signatures of compounds interacting within the enzyme S_1_ pocket. Specifically, the binding of a compound in which the benzamidine anchor has been removed is accompanied by expulsion of one water molecule, a favorable enthalpic event that enhances the overall binding affinity. These results set the stage for development of several orally available anticoagulants designed on the basis of important thermodynamic findings [183]. In summary, there is considerable interest regarding the fundamental role of solvent in modulating thermodynamic signatures of ligand–target interactions, information that is not readily gleaned from structural studies. Solvent molecules may play a significant role in binding energetics and therefore represent a major force in driving a ligand to its desired target.

### 7.2. Conformational Impacts: Ligand Preorganization

The combined efforts of structural and energetics studies may facilitate the design of ligands with improved affinities by lowering their entropic cost of association due to immobilization. In this respect, thermodynamic characterization of ligand-target interactions assists in the overall design process by monitoring a decrease in the entropic penalty to identify compounds with enhanced binding energetics. Ligands that adopt bioactive conformations during late-stage optimization by introducing structural constraints may exhibit greater affinity/potency over their flexible counterparts [200]. The rationale for this improvement relates to entropic advantages assigned to rigid versus flexible ligands. Such techniques have been used to design peptide ligands by conformationally constraining their unbound structural freedom [201]. There are numerous approaches to achieve this goal, which are often performed chemically or enzymatically [202,203].

In terms of drug discovery strategies, thermodynamic analysis at late-stage lead optimizations have yielded significant insights into the role of ligand preorganization on interaction affinity and potency of small molecules [200,204]. The ultimate goal is to identify a preferred bound geometry of ligand within the binding site and simulate its conformation in the solution state via chemical modifications. The latter may include intramolecular hydrogen bonds, cyclization, and other means to shift the ensemble of ligand conformations towards a bioactive form. One particular challenge resides in ensuring that the free ligand-populated conformation faithfully reproduces the active bound state in such a manner that both the conformational strain and entropic penalty are alleviated. An example in which such approaches have been applied to small molecules is the class of BACE inhibitors involving introduction of cyclopropane moieties in the ligand molecular structure [205]. The cyclopropane substituted aminopyrimidone-type BACE-1 inhibitors exhibit improved activity relative to a non-restricted ethylene linker compound. These data correlate well with the resultant entropic gain derived from imposition of conformational constraints due to incorporation of the cyclopropane ring.

### 7.3. Impact of Cooperativity on Binding Energetics

**Figure 16:**
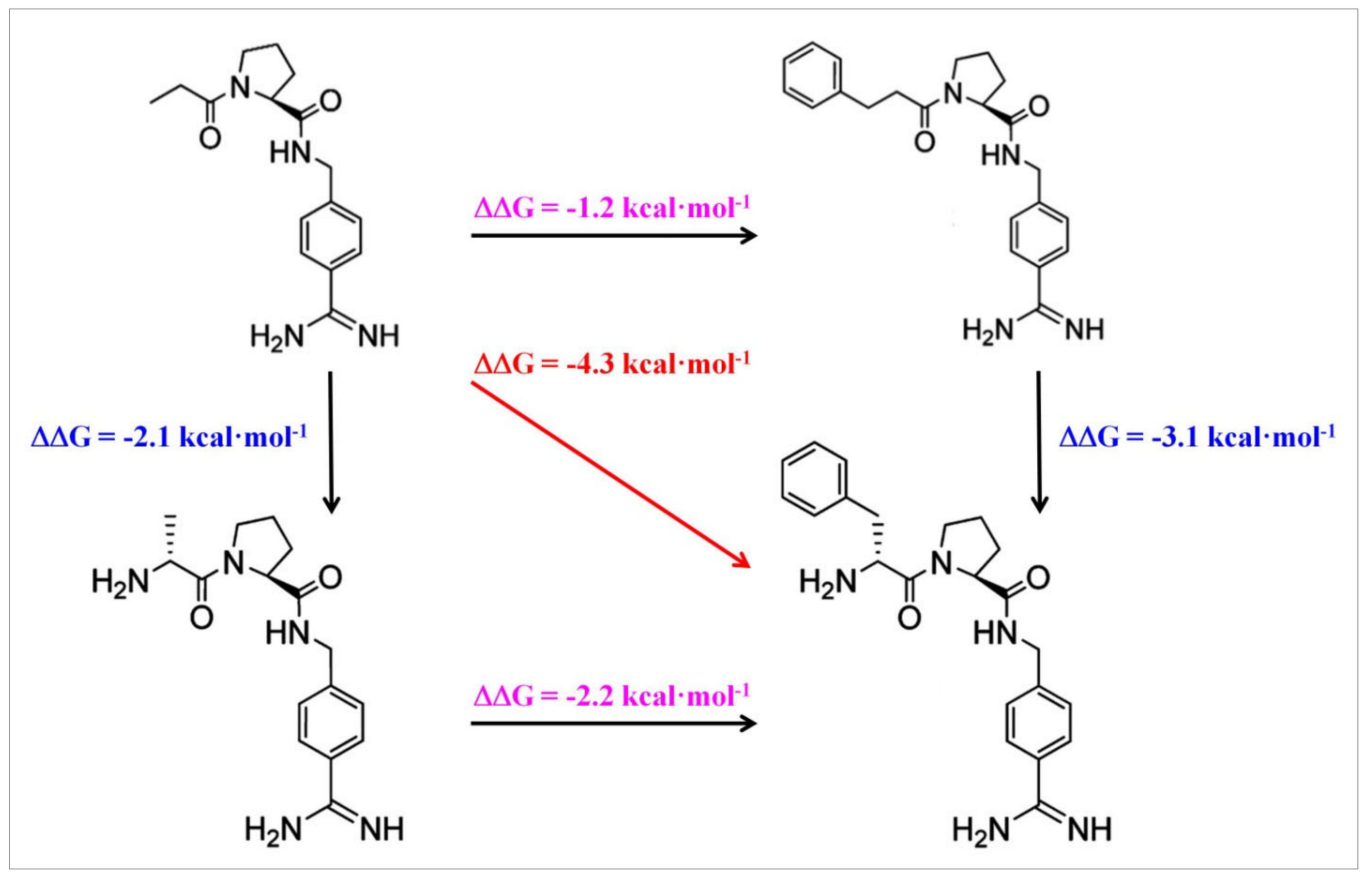
Cooperativity (nonadditivity) of hydrogen bond formation and hydrophobic contacts in a set of thrombin inhibitors analyzed via double replacement cycle. Introduction of a lipophilic side chain (upper horizontal arrow) increases the affinity by 1.2 kcal·mol^−1^. Addition of an amino group (left vertical arrow) increases the affinity by 2.1 kcal·mol^−1^. The combined chemical group substitutions yield a binding free energy enhancement of 4.3 kcal·mol^−1^ (diagonal red arrow) representing a positive cooperativity of ΔΔG = 4.3 − 2.1 − 1.2 = 1.0 kcal·mol^−1^. (Data derived from [208]).

Systematic group substitutions in the ligand [184,185], and/or amino acid replacements in the target protein [186] provide insights into the origins of net thermodynamic binding signatures at the molecular level. The strategy underlying FBDD involves assembling several low affinity fragments with the appropriate geometry and attachment pattern to generate a high affinity ligand that specifically recognizes the target. Deconstruction of the resultant ligand into its respective fragments may or may not yield a binding affinity that reflects the sum of its constituents, which represents a measure of cooperativity [206]. The impact of cooperativity or nonadditivity on binding energetics has been assessed by methyl group substitutions, a popular approach that aims at improving ligand geometry and complementarity in the bound state [207]. The concept of nonadditivity can be appreciated when evaluating congeneric series of compounds [184], where hydrophobic contacts and hydrogen-bond formation between various substituents elicit cooperative effects. A typical example of nonadditivity involving double functional group replacement is presented in Figure 16 for thrombin inhibitors [208]. Inspection of the schematic reveals that single site substitutions of an amino group or benzyl side chain result in binding free energy enhancements (ΔΔG) of −1.2 and −2.1 kcal·mol^−1^, respectively. The simultaneous introduction of both functional groups yields a binding free energy enhancement of −4.3 kcal·mol^−1^, representing a positive cooperativity (i.e., nonadditivity) of ΔΔG = −1.0 kcal·mol^−1^ relative to the sum of individual substitutions (ΔΔG = −3.3 kcal·mol^−1^). It is interesting to note that the order in which group replacements are introduced may play a significant role in the eventual outcome [185].

## 8. Challenges Associated with Interpretation of Thermodynamic Data

The concept of incorporating thermodynamic measurements within the drug discovery arena initially elicited an enthusiastic response that has been tempered by guarded optimism. A notable milestone is the proposal of employing thermodynamic binding signatures as an additional metric in lead optimizations [109], which has been embraced with the expectation that real-time experimental observables might potentially drive the drug development process more effectively. Indeed, such measurements represent a valuable tool for drug development and design, based on the premise that enthalpically driven binders are considered superior ligands in terms of aqueous solubility, decreased toxicity, and higher selectivity [7]. A successful case history that proves this point is illustrated by the various generations of anti-HIV drugs introduced commercially over time, as discussed in Section 3.1 and Section 3.2. Significantly, one observes a general trend in which the newer drugs acquire a thermodynamic signature that gradually shifts from entropy-driven to enthalpically favorable (see Figure 12B).

The most compelling evidence for dissemination of this concept is the recognition that enthalpically driven ligands with polar characteristics retain an added advantage of exhibiting superior pharmacokinetic properties and thereby score additional points in terms of ADMET criteria. As reviewed herein, this paradigm in drug discovery and optimization remains a matter of prioritization in current design strategies yet requires judicious planning and analysis, which integrates rigorous buffer-dependent thermodynamic assessments with high resolution structural capabilities to define binding energetics at the molecular level (refer to additional relevant reviews on this topic [8,209]). Given these constraints and guidelines, contributions arising from solvation, ligand preorganization, cooperativity, and linked processes must be considered explicitly when interpreting thermodynamic signatures within the context of structural data. A clearly defined structural–energetic correlation finally emerges following successful resolution of potential competing events and extrinsic factors that may mask the intrinsic thermodynamic parameters. In this section, we present several caveats associated with the use of thermodynamics in drug discovery strategies and offer suggestions to rationalize some of the unanticipated findings.

### 8.1. Resolving Paradoxes in Thermodynamic Characterizations

Given the increasing use of ITC as an indispensable biophysical tool in drug discovery, coupled with the availability of comprehensive thermodynamic databases, a number of questions have arisen as a consequence of several studies reporting unanticipated outcomes. This prompted serious re-evaluation of available data to corroborate the assumption that *enthalpy-driven binders are favored in drug development* [7]. Additional concerns and queries regarding method protocols, the role of solvation, and impact of ligand conformational constraints [182,210], must be addressed and reassessed [117,211]. Following a period of relative euphoria with the prospect of achieving a successful structural-energetics consensus, a certain level of skepticism has surfaced as drug design laboratories seeking rapid and straightforward decision criteria often face the realization that critical data are lacking for a complete interpretation [7]. One of the caveats associated with employing ITC as a tool in drug development originated as a consequence of the variability observed when conducting measurements under disparate solution conditions, thereby hampering comparison of calorimetric data on an absolute scale. The implementation of rigorous and stringent experimental protocols reduced overall variability and ensured systematic evaluations to resolve intrinsic thermodynamic binding parameters for ligand-target interactions [138,212,213,214].

While ITC is a model-independent technique that provides a direct measure of the heat absorbed/released upon ligand–target association, the resultant enthalpy may be the result of multiple events that occur concomitantly to the binding process. The latter may include and is not restricted to coupled protonation/deprotonation reactions [105,215], and binding-induced conformational changes amongst a host of heat absorbing/releasing events [216]. These linked processes require additional measurements to resolve intrinsic thermodynamic parameters [189,217]. In this respect, ITC protocols must be designed to derive the requisite intrinsic thermodynamic data by conducting measurements in an array of buffers with distinct ionization enthalpies at various pH. The calorimetric experiments should be conducted over a sufficiently broad temperature range to account for heat capacity changes involved in the association process (as reviewed in [131]).

The finding that binding parameters routinely measured in drug discovery campaigns generally do not represent intrinsic thermodynamic signatures has created some reluctance in applying ITC for time-sensitive lead optimizations and drug development [182]. A range of experimental parameters, including temperature and solution conditions (e.g., buffer, solute effects), can significantly influence the observed thermodynamic signatures and must be explicitly considered to derive intrinsic binding enthalpies. Moreover, structural flexibility and allosteric mechanisms may lead to obfuscation in the intrinsic thermodynamic parameters, rendering the latter experimentally inaccessible. The utility of thermodynamics as a core metric to evaluate the potential for success and accelerate drug discovery efforts has been enthusiastically embraced. Nevertheless, a number of unforeseen challenges have hampered the desire to adopt this technique as a routine strategy. A critical review of specific intricacies associated with the use of ITC in drug discovery [182] suggests that while enthalpy and entropy should not be viewed as direct end points, the latter may significantly enhance our understanding of ligand–target interactions when employed in conjunction with structural and/or computational approaches.

### 8.2. Caveats Associated with the Design of a Constrained Ligand

Ligand preorganization may represent a useful strategy in thermodynamic approaches designed to improve binding affinities by reducing entropic penalties associated with conformational strain (Refer to Section 7.2). Considering its potential utility, several unanticipated experimental outcomes led to the realization that additional factors must be considered when designing a “bioactive conformation”. As a case in point, studies on Grb2 SH2 domain-peptide interactions [201] reveal that designed ligands adopting a bound-like geometry are characterized by unexpected improvements in binding enthalpies yet exhibit unfavorable entropic contributions relative to the original flexible conformers [200,211]. These findings suggest a counterproductive outcome when attempting to improve ligand potency by purposely avoiding entropic penalties due to binding-induced conformational strain. A plausible explanation for this apparent discrepancy has been offered by assessing the conformational energetics computationally [117]. Specifically, the conformationally-constrained ligands might further reduce binding site residue entropies, thereby offering a counterargument to the expected improvement in overall binding entropy upon ligand pre-organization.

In the final analysis, thermodynamic binding signatures must explicitly consider multiple factors and parameters in order to enable reliable predictions. Along these lines, the inventory of water molecules involved in a ligand-target interaction is an important consideration [200,211], as solvation plays a fundamental role in the binding energetics and overall enthalpy/entropy balance. The general consensus is to introduce some constraints in the small molecule that create a *pre-organized state*, which is poised to interact with the binding site while introducing no major strain. In principle, this represents a reasonable approach yet requires a number of important assumptions, namely: (a) the constrained ligand conformation is equivalent to the final bound state; and, (b) hydration and hydrogen bond donor/acceptor capabilities are intact. Under these conditions, the resultant energetic signatures are characterized by an entropy gain and enhanced binding affinity. In practice, there are systems in which the entropy gain is balanced by an enthalpy loss resulting in marginal improvement of the binding affinity. Conceivable origins for such unexpected outcomes associated with conformationally constrained ligands may reside in violation of specific conditions related to the integrity of hydration and/or hydrogen bond donor/acceptor capabilities as a consequence of ligand design.

### 8.3. Origins of Enthalpy-Driven Hydrophobic Interactions

The well described hydrophobic effect suggests that the burial of non-polar surfaces represents an entropically favorable process, implying that addition of non-polar residues to small molecules enhances the binding affinity via entropy gain. Although typically viewed as an entropy-driven process, examples of hydrophobic interactions that are enthalpic in nature have been documented in a comprehensive review on this topic [218]. There are several plausible explanations for enthalpy-driven hydrophobic effects including binding-induced release of water molecules from the hydrophobic environment to bulk water [219]. Conversely, an opposing entropic component arises due to the elimination of solvent fluctuations inside the binding pocket. Such contributions override the favorable entropic effects of extracting a small non-polar ligand from bulk solvent.

Water molecules that form non-optimal hydrogen-bonds within a hydrophobic surface are released upon ligand binding and optimally hydrogen-bonded in the bulk solvent which is an enthalpically favorable process. Another possibility arises when the binding pocket of an apo protein is suboptimally hydrated. In such situations, solute-solvent dispersion interactions in the hydrated complex might not be completely offset by dispersion interactions for the hydrated protein and ligand. The concomitant increase in dispersive interactions upon complexation may therefore result in a favorable enthalpic contribution. An excellent example is the interaction between major urinary protein (MUP-1) and primary alcohols of various chain length in which a combination of calorimetric, structural, and computational studies reveal that the association process is enthalpy-driven, despite the hydrophobicity of interacting species [220]. The authors rationalize these findings as conceivably arising from favorable solute-solute dispersion interactions following protein–ligand complexation. Indeed, the apo-MUP-I pocket is suboptimally hydrated [221], which implies an inequality between solute–solvent dispersion interactions prior to protein–ligand binding versus solute–solute dispersion interactions in the associated complex.

**Figure 17:**
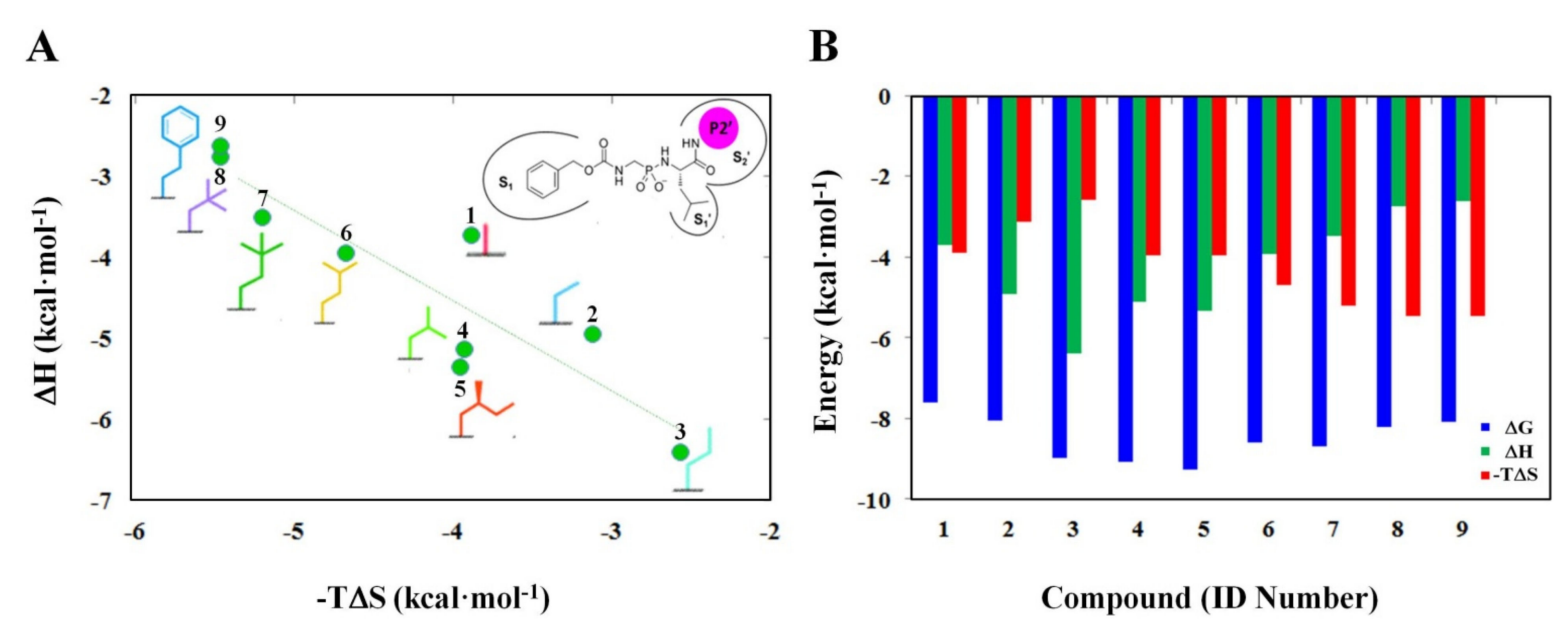
(**A**) Enthalpy-entropy plot for a congeneric series of thermolysin inhibitors harboring distinct substitutions at the P_2′_ position with each compound identified according to entry number (**1**–**9**) and respective group substituent; (**B**) Thermodynamic binding signatures [i.e., ΔG (blue), ΔH (green), and −TΔS (red)] for the thermolysin inhibitors presented in Panel A. Ligands with medium-sized P_2_′ substituents (i.e., compounds **4** and **5**) exhibit the highest affinities as a consequence of favorable enthalpy and entropy contributions. (Data derived from [222]).

Several studies involving congeneric ligands highlight some of the challenges associated with understanding how adding nonpolar surface area to small molecules affects their protein-binding energetics. An intriguing example in which increased non-polar surface area modulates the thermodynamic binding signature is illustrated in Figure 17 for a series of thermolysin inhibitors [222]. These enzyme inhibitors have been designed via P_2′_ substitutions, as noted in the inset of Figure 17A. Inspection of the enthalpy-entropy plot in Figure 17A and resultant thermodynamic signatures sorted by molecular size in Figure 17B reveals that while compounds **1**–**3** exhibit an increased enthalpic contribution as a function of size, ligands **4**–**5** are virtually indistinguishable thermodynamically, and compounds **6**–**9** exhibit the expected size-dependent enhancement in binding entropy as deduced from increased surface area burial. In the latter group, entropy gains are balanced by reduced enthalpic interactions, which is consistent with a structural loss of definition and increased motion in some complexes. An overall evaluation of the enthalpy/entropy balance reveals that whereas enthalpy prevails for smaller substituents, the entropic component dominates ΔG for larger substituents. It is interesting to note that ligands with medium-sized P_2′_-substituents (i.e., compounds **4** and **5**) exhibit the highest affinities. In an effort to rationalize these observations, inspection of the water geometries adjacent to P_2′_ via X-ray crystallography [222] suggests correlation between an optimal solvation network and the resultant thermodynamic profiles. These findings underscore the importance of performing systematic evaluations within congeneric compounds to establish correlations between structural and energetic properties, as useful information can be gleaned from such comparisons [223], including the paradoxical nature of enthalpically driven hydrophobic interactions.

## 9. Potential of Structure-Energetic Correlations in Accelerating Drug Design Predictions

Elucidation of biomolecular interactions at the atomic level is extremely complex as these involve formation and/or disruption of multiple non-covalent bonds between the interacting molecules as well as solvent [113]. Despite several decades of experimental studies and extensive analyses, a complete understanding of the thermodynamic driving forces governing biomolecular interactions remains elusive. While ITC provides the most accurate and reliable experimental technique to achieve a complete thermodynamic characterization, the utility of such information in drug design and development still represents a challenge. Correlations between thermodynamic data and structural features yield invaluable insights on biomolecular interactions and computational tools have empowered such correlations at the molecular level. Incorporation of multiparametric approaches in drug discovery strategies represents a powerful infrastructure for the development of future treatment regimens exhibiting enhanced efficacy. Thermodynamics can still provide the requisite input into decision-making processes as the goal of rational drug design is to identify small molecule substitutions that increase compound efficiency, potency, and specificity while minimizing overall toxicity. Thermodynamics may assist in this endeavor by gauging the progress achieved or drawbacks encountered when certain molecular manipulations tend to elicit an unfavorable response. Specifically, characterization of the binding energetics associated with chemical modifications/substitutions facilitates assessment of group-group interactions, conformational constraints, and accommodation within the binding site. The resultant thermodynamic signature in conjunction with a high-resolution structure furnishes a complete molecular description of biomolecular interactions within the ligand-target complex.

There is a fundamental need to expand predictive capabilities based on experimental structural-energetics data that inform decisions regarding the selection of hits worth pursuing at the next level of drug design and development. In order to identify the most promising candidates for lead optimization, predictive biophysical parameters are required, and thermodynamic data can furnish valuable insights to achieve this goal. Approaches towards this direction have been pursued and score functions derived from structural and calorimetric data [224]. ITC experiments provide direct access to ΔG, ΔH, TΔS and ΔH/TΔS compensation as a function of specific experimental variables including buffer, temperature, osmolytes, and pH [188]. Resolving intrinsic thermodynamic parameters is therefore essential for an unbiased structural-energetic analysis. While the current review mines available thermodynamic databases as an illustration of overall trends and provides insights on the interplay between experimental observables and design metrics, it is highly advisable to evaluate differential binding profiles across specific data sets comprising congeneric ligand series for precise interpretations [223]. Such systematic evaluations have proven particularly illuminating by resolving apparent paradoxes and gaining a more detailed understanding of the forces driving specific ligand target interactions at both the structural and thermodynamic levels.

A considerable body of evidence has demonstrated that ligand preorganization abrogates the entropic penalty associated with structural rearrangements. Conversely, entropically favorable interactions arising from residual mobility of ligand or target in the bound state is often compensated by an unfavorable enthalpic term. In most cases, desolvation is an entropically favorable process, yet there are instances in which such interactions are enthalpically favored. An unexpected observation is that the role of local water structure may dramatically alter the thermodynamic binding signatures without any appreciable impact on ligand affinity. These findings underscore the need to evaluate biomolecular interactions thermodynamically, as characterization of binding affinities alone may miss important molecular events that are only distinguished in the enthalpic and/or entropic terms. Intrinsic thermodynamic binding parameters should be correlated with high resolution crystallographic data, as low resolution structures lack fundamental aspects related to water inventory, thereby precluding optimal structural-energetic correlations at atomic resolution [7].

Despite the challenges of correlating specific fragment substitutions and design changes in small molecules with their respective thermodynamic binding profiles, the latter provide additional layers of valuable information to elucidate the forces governing specific ligand-target interactions. Computational studies aimed at complementing these assessments rely on experimental databases that are incomplete yet necessary to correlate specific biophysical properties at a molecular level with the net thermodynamic consequences of chemical modifications/substitutions. A critical review of progress achieved spanning three decades suggests that there is a fundamental role for thermodynamics in drug discovery strategies. Although thermodynamic signatures might not necessarily constitute endpoints for lead optimizations such as cases in which a multitude of complex events hamper resolution of the driving forces [225], thermodynamic profiling combined with high resolution structural data represent an enormous asset to accelerate drug development and optimization [182]. The collective efforts of dedicated thermodynamicists, structural biologists, and computational scientists armed with the requisite biomolecular data and biophysical parameters should improve overall predictive capabilities whereby structure informs biology and energetics provides the foundation for decision making on hit-to-lead optimizations in drug design and development.

## 10. Concluding Remarks

This manuscript presents an overview of experimental approaches employed in drug discovery strategies to identify and develop prospective molecules for further optimization as lead compounds in treatment regimens. Particular emphasis is focused on the fundamental role of thermodynamics in drug design and optimization by applying calorimetric methods to characterize the forces driving ligand-target association processes in solution. Considering the relevance of utilizing direct model-independent calorimetric data to elucidate macromolecular binding energetics, ongoing efforts are concentrated on the development of high-throughput methodologies aimed at deriving rapid yet accurate thermodynamic parameters while employing minimal quantities of biomaterials. The necessity and urgency of devising novel technological approaches to evaluate the biological/biophysical properties of lead compounds for the express purpose of optimizing their efficacy in terms of bioavailability, potency, and specificity is readily apparent. Drug discovery technologies rely on high throughput screenings whereby hit-to-lead decisions and compound optimization inevitably requires validations by combining structural and functional methodologies. Energetics-based approaches successfully bridge the gap between structure and function as calorimetric data fill the void by furnishing a complete description of biomolecular interactions via the elucidation of thermodynamic binding signatures and driving forces.

## 11. Dedication

The authors express their grateful and heartfelt appreciation to Professor Kenneth J. Breslauer, a distinguished mentor and lifelong friend, who has served as an exemplary role model throughout our scientific careers. In recognition of his seventh-fifth birthday celebration, we dedicate this manuscript as a personal tribute with our deepest admiration, affection, gratitude, and respect.

## Data Availability

Not Applicable.

## Abbreviations

**ADMET**	Absorption, Distribution, Metabolism, Excretion, Toxicity
**AlogP**	Computationally Derived LogP
**BEI**	Binding Efficiency Index
**BLI**	Biolayer Interferometry
**bRo5**	Beyond Rule of Five
**CE**	Capilary Electrophoresis
**ClogP**	Computationally Derived LogP
**CryoEM**	Cryo-Electron Microscopy
**DSF**	Differential Scanning Fluorimetry
**EE**	Enthalpic Efficiency
**FBDD**	Fragment-Based Drug Discovery
**GCI**	Grating-Coupled Interferometry
**GE**	Group Efficiency
**HA**	Heavy Atoms
**HBA**	Hydrogen Bond Acceptors
**HBD**	Hydrogen Bond Donors
**HTS**	High Throughput Screening
**IDP**	Intrinsically Disordered Protein
**ITC**	Isothermal Titration Calorimetry
**LE**	Ligand Efficiency
**LipE**	Lipophilic Efficiency
**LLE**	Ligand Lipophilic Efficiency
**LogP**	Logarithm of Octanol/Water Partition Coefficient
**MST**	Microscale Thermophoresis
**NME**	New Molecular Rntities
**NMR**	Nuclear Magnetic Resonance
**NNH**	Number of non-hydrogen atoms
**NP**	Natural Products
**PAINS**	Pan-Assay Interference Compounds
**PSA**	Polar Surface Area
**QSAR**	Quantitative Structure-Activity Relationships
**Ro3**	Rule of Three
**Ro5**	Rule of Five
**ROTB**	Rotatable Bonds
**SBDD**	Structure-Based Drug Design
**SIHE**	Size Independent Enthalpic Efficiency
**SPR**	Surface Plasmon Resonance
**TPSA**	Topological Polar Surface Area
**TSA**	Thermal Shift Assay
**WAC**	Weak Affinity Chromatography
